# Supplementary material for: Proteomic Learning of Gamma-Aminobutyric Acid (GABA) Receptor-Mediated Anesthesia
Source: J Chem Inf Model. 2025 Mar 17;65(7):3655–68. doi: 10.1021/acs.jcim.5c00114 (PMC12004937; doi:10.1021/acs.jcim.5c00114)
Supplement: Supplementary file 1 — ci5c00114_si_001.pdf [file ci5c00114_si_001.pdf]

Supporting Information: Proteomic Learning of Gamma-Aminobutyric Acid (GABA) Receptor-Mediated Anesthesia

Jian Jiang[1,2,*], Long Chen[1], Yueying Zhu[1], Yazhou Shi[1], Huahai Qiu[1], Bengong Zhang[1], Tianshou Zhou[3], and Guo-Wei Wei[2,4,5,+]

[1]Research Center of Nonlinear Science, School of Mathematical and Physical Sciences, Wuhan Textile University, Wuhan, 430200, P R. China

[2]Department of Mathematics, Michigan State University, East Lansing, Michigan 48824, USA

[3]Key Laboratory of Computational Mathematics, Guangdong Province, and School of Mathematics, Sun Yat-sen University, Guangzhou, 510006, P R. China

[4]Department of Electrical and Computer Engineering Michigan State University, East Lansing, Michigan 48824, USA

[5]Department of Biochemistry and Molecular Biology Michigan State University, East Lansing, Michigan 48824, USA

[*]Corresponding author: jjiang@wtu.edu.cn

[+]Corresponding author: weig@msu.edu

S1 Datasets, performance summary and PPI networks

S1.1 Datasets and performance summary

In this work, we extracted 24 PPI networks centered on GABA receptors from the String database. To construct binding affinity prediction models for proteins in the PPI networks, we collected inhibitor datasets from the ChEMBL database (https://www.ebi.ac.uk/chembl/). We also collected inhibitor datasets for the hERG potassium channel from ChEMBL. This resulted in a total of 136 datasets. Table S1 summarizes the information about these datasets. The predictive performance of our model is demonstrated by the Pearson correlation coefficients (R) and root-mean-square deviation (RMSD) (kcal/mol) in the ten-fold cross-validation test, listed in the last two columns of Table S1. Note that the first column in Table S1 records the names of the collection datasets for the corresponding proteins for which we want to construct machine-learning models. The abbreviations are derived from these protein names. The full names of the proteins are listed in Table S6. The distribution of all 136 datasets is shown in Figures S3, S4, S5, and S6. The 24 PPI networks for GABA receptors are presented in Figures S7 and S8.

**Dataset**	**ChEMBL ID**	**Dataset size**	**Binding affinity range(kcal/mol)**	**R of 10-fold CV**	**RMSD of 10-fold CV(kcal/mol)**
GABRA5	CHEMBL5112	477	[-13.63,-7.23]	0.788	0.634
AAK1	CHEMBL3830	248	[-14.25,-5.6]	0.768	1.042

ADORA1	CHEMBL226	4514	[-14.77,-5.62]	0.789	0.854
ADORA3	CHEMBL256	4254	[-15.0,-1.45]	0.86	0.87
ADRA2A	CHEMBL1867	914	[-14.15,-5.46]	0.807	0.995
ADRB1	CHEMBL213	849	[-14.59,-5.59]	0.814	0.929
ADRB2	CHEMBL210	1033	[-14.89,-5.24]	0.841	1.046
ADRB3	CHEMBL2468	376	[-12.27,-5.5]	0.678	0.938
AGTR2	CHEMBL4607	462	[-14.18,-5.9]	0.779	0.956
AKT1	CHEMBL4282	3192	[-14.04,-5.69]	0.857	0.842
APLNR	CHEMBL1628481	275	[-14.86,-6.03]	0.808	1.233
ATG4B	CHEMBL1741221	419	[-10.68,-5.88]	0.654	0.493
ATG7	CHEMBL2321621	199	[-10.91,-5.86]	0.546	0.468
BCL2L1	CHEMBL4625	1069	[-14.35,-4.96]	0.909	0.827
BDKRB2	CHEMBL3157	515	[-14.45,-5.79]	0.874	1.008
CACNA1B	CHEMBL4478	379	[-11.51,-5.86]	0.823	0.569
CACNA1C	CHEMBL1940	183	[-13.09,-5.86]	0.763	0.963
CACNA1G	CHEMBL4641	543	[-12.68,-6.02]	0.852	0.727
CACNA1H	CHEMBL1859	410	[-11.96,-5.89]	0.81	0.716
CACNA1I	CHEMBL5558	250	[-12.84,-6.82]	0.834	0.831
CALCR	CHEMBL1832	129	[-15.41,-7.49]	0.497	1.271
CALCRL	CHEMBL3798	735	[-15.41,-5.89]	0.883	1.017
CASP3	CHEMBL233	4889	[-15.35,-5.35]	0.842	0.951
CASP8	CHEMBL3776	341	[-15.13,-5.59]	0.809	1.031
CCKAR	CHEMBL1901	437	[-15.41,-6.48]	0.865	1.102
CCR3	CHEMBL3473	1133	[-13.94,-5.73]	0.892	0.784
CCR4	CHEMBL2414	495	[-13.36,-5.86]	0.784	0.85
CHRM1	CHEMBL216	1830	[-14.25,-5.45]	0.833	1
CHRM2	CHEMBL211	1816	[-15.0,-5.48]	0.867	0.985
CHRM3	CHEMBL245	2097	[-14.73,-5.47]	0.9	0.971
CHRM4	CHEMBL1821	928	[-14.59,-5.45]	0.849	0.971
CHRNA7	CHEMBL2492	615	[-14.35,-5.45]	0.792	1.092
CNR1	CHEMBL2182	78	[-13.89,-5.45]	0.605	0.7
CNR2	CHEMBL253	4503	[-14.8,-5.48]	0.839	0.867
CTSB	CHEMBL4072	1292	[-14.78,-5.45]	0.8	0.989
CTSD	CHEMBL2581	1554	[-14.94,-5.59]	0.825	0.882
CTSL	CHEMBL3837	1900	[-14.45,-5.45]	0.814	1.006
CTSS	CHEMBL2954	2229	[-14.78,-5.68]	0.874	0.868
CXCR2	CHEMBL2434	1009	[-14.04,-6.58]	0.806	0.889
DAPK3	CHEMBL246	323	[-12.27,-5.45]	0.802	0.847
DRD1	CHEMBL2056	1173	[-13.6,-5.88]	0.819	0.862
DRD2	CHEMBL217	7600	[-14.41,-5.1]	0.801	0.852
DRD3	CHEMBL234	5029	[-14.59,-5.64]	0.845	0.861
DRD4	CHEMBL219	2658	[-14.65,-5.52]	0.775	0.915
DRD5	CHEMBL1850	333	[-13.99,-5.56]	0.857	0.894
ERBB4	CHEMBL3009	293	[-13.66,-6.6]	0.792	0.979
ERN1	CHEMBL1163101	354	[-14.45,-6.46]	0.734	0.847
GCGR	CHEMBL1985	1099	[-14.04,-5.48]	0.829	0.735
GHSR	CHEMBL4616	1344	[-15.0,-5.86]	0.731	1.023
GLRA1	CHEMBL5845	58	[-10.32,-6.66]	0.74	0.645
GRIK1	CHEMBL1918	199	[-12.45,-5.69]	0.67	1.009

GRIN2B	CHEMBL1904	220	[-12.48,-5.93]	0.646	1.001
GRM1	CHEMBL3772	648	[-13.22,-5.54]	0.822	0.922
GRM2	CHEMBL5137	784	[-12.62,-5.56]	0.855	0.634
GRM3	CHEMBL2888	133	[-12.72,-5.84]	0.744	0.933
GRM4	CHEMBL2736	48	[-11.99,-5.47]	0.884	1.088
GRM5	CHEMBL3227	1777	[-13.23,-5.47]	0.751	0.88
HAP1	CHEMBL5619	177	[-10.31,-5.86]	0.707	0.711
HCAR2	CHEMBL3785	312	[-12.68,-5.86]	0.802	0.83
HCRTR2	CHEMBL4792	3119	[-14.59,-6.77]	0.826	0.781
HDAC6	CHEMBL1865	4289	[-14.04,-5.86]	0.848	0.799
HIF1A	CHEMBL4261	394	[-12.7,-5.61]	0.762	0.852
HSP90AA1	CHEMBL3880	1174	[-12.92,-5.89]	0.843	0.657
HTR1A	CHEMBL214	4684	[-14.85,-5.53]	0.831	0.861
HTR1B	CHEMBL1898	1090	[-23.65,-6.48]	0.798	1.037
HTR1D	CHEMBL1983	1266	[-14.59,-5.85]	0.894	0.839
HTR1E	CHEMBL218	4016	[-11.18,-6.82]	0.845	0.87
HTR2A	CHEMBL224	4749	[-15.0,-5.45]	0.838	0.841
HTR2C	CHEMBL225	3055	[-14.59,-5.45]	0.781	0.869
HTR4	CHEMBL1875	468	[-14.72,-6.27]	0.866	0.921
HTR5A	CHEMBL3426	317	[-12.68,-5.73]	0.791	0.932
HTR6	CHEMBL3371	4092	[-14.18,-5.62]	0.865	0.782
HTR7	CHEMBL3155	2648	[-13.63,-5.45]	0.804	0.834
KCNK3	CHEMBL2321613	535	[-12.68,-5.88]	0.662	0.678
KEAP1	CHEMBL2069156	259	[-12.07,-5.86]	0.753	1.04
LPAR1	CHEMBL3819	228	[-13.39,-6.63]	0.757	0.851
LTB4R	CHEMBL3911	343	[-14.04,-5.45]	0.766	1.019
MAOB	CHEMBL2039	4142	[-13.87,-5.05]	0.81	0.962
MCL1	CHEMBL4361	2356	[-14.89,-4.65]	0.874	0.665
MRGPRX4	CHEMBL4523430	203	[-11.59,-8.59]	0.535	0.624
MTNR1A	CHEMBL1945	1035	[-15.27,-5.63]	0.827	1.062
MTNR1B	CHEMBL1946	927	[-15.0,-5.71]	0.806	1.053
MTOR	CHEMBL2842	4417	[-14.25,-5.86]	0.877	0.748
NLRP3	CHEMBL1741208	273	[-11.73,-6.04]	0.778	0.716
NOD2	CHEMBL1293266	220	[-12.68,-6.22]	0.748	0.785
NPY1R	CHEMBL4777	890	[-15.0,-5.53]	0.855	0.949
NPY2R	CHEMBL4018	656	[-14.25,-5.88]	0.883	0.931
NTRK2	CHEMBL4898	662	[-14.04,-5.45]	0.849	1.011
NTSR1	CHEMBL4123	249	[-15.0,-5.57]	0.779	1.406
OPRD1	CHEMBL236	4191	[-15.0,-5.46]	0.86	0.938
OPRM1	CHEMBL2334	1857	[-15.27,-5.6]	0.915	0.781
OXTR	CHEMBL2049	693	[-14.45,-5.86]	0.767	0.976
P2RY12	CHEMBL2001	1101	[-13.36,-6.02]	0.852	0.761
PIK3C3	CHEMBL1075165	160	[-13.09,-6.41]	0.53	1.128
PIK3CB	CHEMBL3145	2138	[-14.45,-5.71]	0.852	0.857
PIK3CD	CHEMBL3130	3408	[-14.59,-5.86]	0.835	0.888
PIK3CG	CHEMBL3267	2850	[-15.0,-4.55]	0.823	0.862
PKMYT1	CHEMBL3984	99	[-11.88,-6.6]	0.726	1.02
PRKAA1	CHEMBL4045	331	[-12.95,-5.45]	0.65	0.915
PRKCE	CHEMBL3582	407	[-12.89,-5.88]	0.72	1.056

PRLHR	CHEMBL1681611	162	[-11.62,-7.04]	0.713	0.519
PTGER1	CHEMBL1811	740	[-13.89,-5.55]	0.848	0.876
PTGER2	CHEMBL1881	361	[-12.21,-5.55]	0.786	0.841
PTGER3	CHEMBL3710	707	[-13.17,-5.45]	0.861	0.809
PTGER4	CHEMBL1836	694	[-14.59,-5.94]	0.825	0.997
RIPK1	CHEMBL5464	279	[-13.57,-6.24]	0.585	1.119
RIPK2	CHEMBL5014	227	[-15.35,-6.0]	0.697	1.047
RPS6KB1	CHEMBL4501	1815	[-14.35,-5.97]	0.868	0.827
S1PR1	CHEMBL4333	752	[-15.41,-6.09]	0.864	0.997
S1PR2	CHEMBL2955	303	[-12.81,-6.59]	0.854	0.762
S1PR3	CHEMBL3892	375	[-13.36,-6.19]	0.802	0.908
S1PR4	CHEMBL3230	272	[-12.62,-7.23]	0.803	0.672
SCN10A	CHEMBL5451	242	[-13.39,-5.88]	0.839	0.903
SCN4A	CHEMBL2072	239	[-14.33,-5.58]	0.684	0.959
SCN5A	CHEMBL1980	976	[-13.43,-5.86]	0.712	0.715
SCN9A	CHEMBL4296	5911	[-15.08,-5.93]	0.843	0.725
SIRT1	CHEMBL4506	673	[-12.89,-5.86]	0.813	0.774
SIRT3	CHEMBL4461	233	[-11.99,-5.92]	0.799	0.84
SLC1A1	CHEMBL2721	106	[-10.36,-5.5]	0.652	0.904
SLC1A2	CHEMBL4973	115	[-11.16,-5.65]	0.83	0.787
SLC1A3	CHEMBL3085	146	[-12.13,-5.5]	0.701	0.961
SLC6A1	CHEMBL1903	96	[-10.67,-5.87]	0.707	0.971
SLC6A3	CHEMBL238	2757	[-14.45,-5.49]	0.838	0.828
SLC6A4	CHEMBL228	4519	[-15.0,-5.64]	0.83	0.868
SLC6A5	CHEMBL222	3158	[-14.45,-5.49]	0.825	0.846
SSTR1	CHEMBL1917	435	[-13.66,-6.54]	0.804	0.866
SSTR2	CHEMBL1804	658	[-15.0,-5.63]	0.854	1.004
SSTR3	CHEMBL2028	664	[-13.32,-6.35]	0.768	0.909
SSTR4	CHEMBL1853	509	[-13.09,-5.66]	0.882	0.835
SSTR5	CHEMBL1792	792	[-13.89,-6.09]	0.862	0.86
STK3	CHEMBL4708	396	[-12.82,-6.58]	0.656	0.819
TAS2R8	CHEMBL3988599	320	[-11.32,-6.88]	0.777	0.584
TBK1	CHEMBL5408	488	[-14.35,-5.59]	0.727	0.985
USP30	CHEMBL4523357	451	[-12.68,-7.23]	0.652	0.636
VCP	CHEMBL1075145	404	[-11.53,-6.27]	0.771	0.739
hERG	CHEMBL240	9151	[-13.84,-5.55]	0.778	0.77

Table S1: The summary of 136 inhibitor datasets used in this study.

Property	Optimal range
FDAMDD	Excellent: 0-0.3; medium: 0.3-0.7; poor: 0.7-1.0
$F_{20\%}$	Excellent: 0-0.3; medium: 0.3-0.7; poor: 0.7-1.0
Log P	The proper range: 0-3 log mol/L
Log S	The proper range: -4-0.5 log mol/L
$T_{1/2}$	Excellent: 0-0.3; medium: 0.3-0.7; poor: 0.7-1.0
Caco-2	The proper range: >-5.15
SAS	The proper range: <6

Table S2: The optimal ranges of six selected ADMET properties and synthesizability (SAS) used to screen nearly optimal compounds.

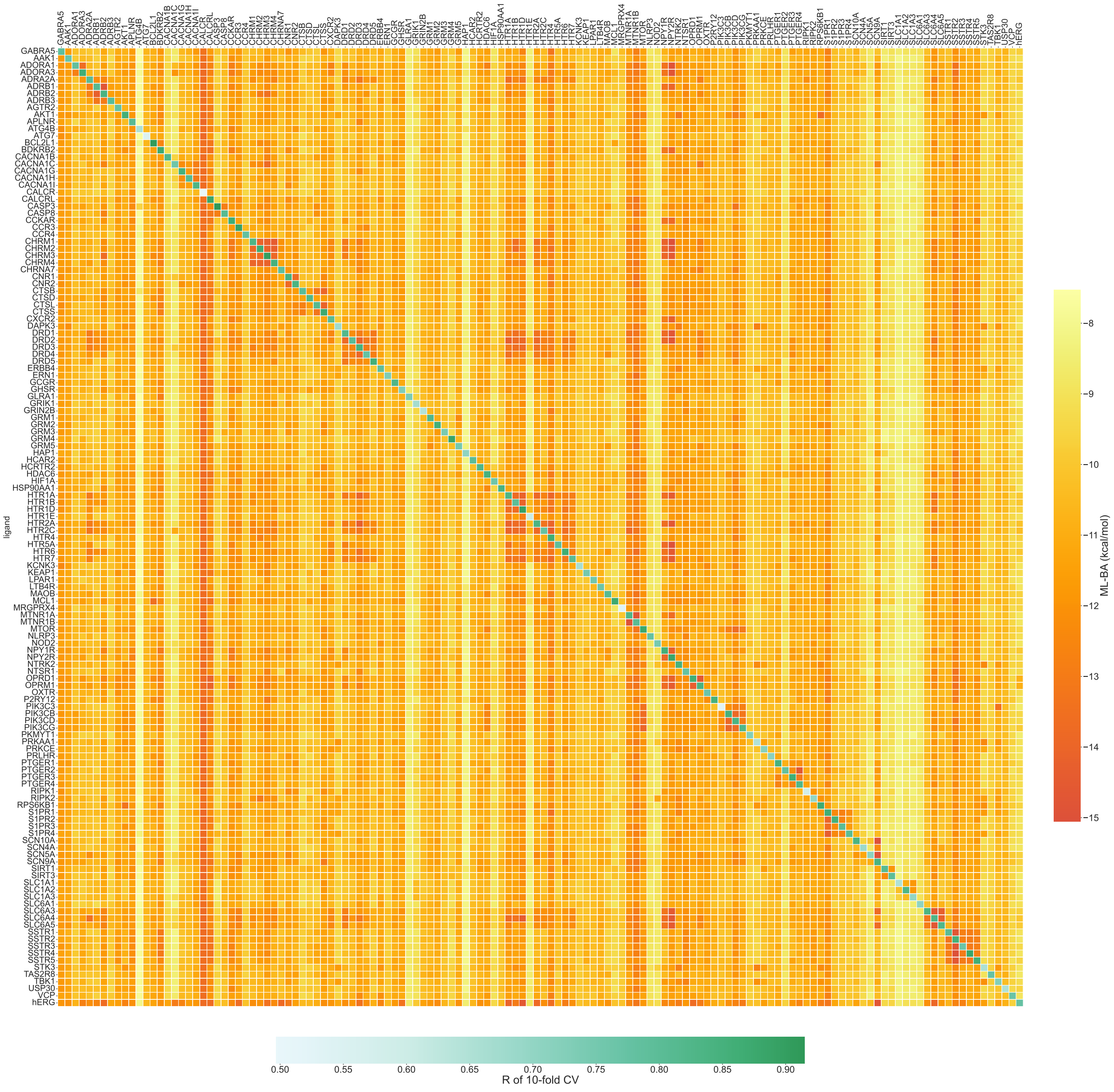

Figure S1: The heatmap for cross-target BA predictions reveals the inhibitor specificity of each dataset to various protein targets. The symbols above the heatmap represent the ML models, while the symbols on the left indicate the inhibitor datasets. The diagonal elements in the heatmap represent the Pearson correlation coefficient (R) from the ten-fold cross-validation of all prediction models. The off-diagonal elements in each row show the highest BA value predicted for the inhibitors in one dataset by the 136 ML models.

S1.2 Detailed information on model construction and performance comparison

The hyperparameters used to construct 136 machine learning predictors or for modeling by ten-fold cross-validation tests are shown in Table S3. We compared the three models, Support Vector Machine (SVM), Gradient Boosting Decision Trees (GBDT), and Random Forest (RF), through the ten-fold cross-validation test. The results of the comparison are recorded in Table S4, and by observation, we found that SVM performs better than GBDT and RF on most of the datasets. Meanwhile, the use of a consensus strategy leads to better performance and more effective machine learning compared to the bidirectional encoder transformer (BET) and autoencoder (AE) models that extract features individually.

Table S5 shows the comparison of the predictions of the two NLP fingerprints and their consensus results on 136 datasets. It is observed that the BET fingerprint outperforms the AE fingerprint in terms of mean R and RMSD values. The average R and RMSD of BET are 0.779 and 0.900 respectively, whereas the average R and RMSD of AE are 0.775 and 0.905 respectively. In contrast, using the consensus model improves the performance even further with an average R and RMSD of 0.788 and 0.883 respectively.

Train Size	RF Parameters	GBDT Parameters	SVM Parameters
<1000	n_estimators=10000<br>criterion='mse'<br>max_depth=7<br>min_samples_split=3<br>min_samples_leaf=1	n_estimators=10000<br>max_depth=7<br>min_samples_split=3<br>subsample=0.7<br>learning_rate=0.01<br>max_features='sqrt'	C=10<br>gamma='scale'<br>kernel='rbf'
[1000,5000)	n_estimators=10000<br>criterion='mse'<br>max_depth=8<br>min_samples_split=4<br>min_samples_leaf=2	n_estimators=10000<br>max_depth=8<br>min_samples_split=4<br>subsample=0.5<br>learning_rate=0.01<br>max_features='sqrt'	C=5<br>gamma='scale'<br>kernel='rbf'
>5000	n_estimators=10000<br>criterion='mse'<br>max_depth=9<br>min_samples_split=7<br>min_samples_leaf=3	n_estimators=10000<br>max_depth=9<br>min_samples_split=7<br>subsample=0.3<br>learning_rate=0.01<br>max_features='sqrt'	C=1<br>gamma='scale'<br>kernel='rbf'

Table S3: The hyperparameters of RF, SVM, and GBDT for datasets with different training-set sizes. All parameters not covered in the table below use the default values in the algorithm.

Regression	Metrics					
	Average R			Average RMSD		
Dataset	BET	AE	consensus (BET+AE)	BET	AE	consensus (BET+AE)
GABRA5	0.778	0.781	0.788	0.648	0.644	0.634
AAK1	0.767	0.753	0.768	1.043	1.070	1.042
ADORA1	0.777	0.781	0.789	0.875	0.868	0.854
ADORA3	0.853	0.851	0.86	0.888	0.893	0.870
ADRA2A	0.792	0.804	0.807	1.027	1.002	0.995
ADRB1	0.808	0.806	0.814	0.943	0.947	0.929
ADRB2	0.837	0.831	0.841	1.056	1.075	1.046
ADRB3	0.801	0.782	0.802	0.848	0.881	0.847
AGTR2	0.755	0.775	0.779	1.000	0.964	0.956
AKT1	0.848	0.851	0.857	0.866	0.857	0.842
APLNR	0.773	0.799	0.808	1.322	1.255	1.233
ATG4B	0.601	0.654	0.64	0.524	0.493	0.501

ATG7	0.511	0.542	0.546	0.482	0.470	0.468
BCL2L1	0.902	0.901	0.909	0.853	0.856	0.827
BDKRB2	0.858	0.87	0.874	1.065	1.020	1.008
CACNA1B	0.8	0.823	0.823	0.599	0.568	0.569
CACNA1C	0.722	0.746	0.739	0.962	0.926	0.937
CACNA1G	0.844	0.846	0.852	0.745	0.740	0.727
CACNA1H	0.788	0.81	0.809	0.752	0.716	0.719
CACNA1I	0.823	0.824	0.834	0.851	0.851	0.831
CALCR	0.497	0.084	0.357	1.271	1.556	1.364
CALCRL	0.877	0.873	0.883	1.040	1.056	1.017
CASP3	0.91	0.911	0.915	0.801	0.797	0.781
CASP8	0.796	0.808	0.809	1.064	1.034	1.031
CCKAR	0.862	0.849	0.865	1.114	1.158	1.102
CCR3	0.886	0.886	0.892	0.802	0.803	0.784
CCR4	0.771	0.777	0.784	0.874	0.863	0.850
CHRM1	0.826	0.824	0.833	1.019	1.025	1.000
CHRM2	0.856	0.863	0.867	1.020	0.998	0.985
CHRM3	0.892	0.898	0.9	1.004	0.980	0.971
CHRM4	0.838	0.84	0.849	1.001	0.997	0.971
CHRNA7	0.79	0.769	0.792	1.097	1.144	1.092
CNR1	0.836	0.839	0.845	0.894	0.886	0.870
CNR2	0.83	0.831	0.839	0.889	0.888	0.867
CTSB	0.788	0.788	0.8	1.010	1.012	0.989
CTSD	0.821	0.813	0.825	0.892	0.909	0.882
CTSL	0.8	0.811	0.814	1.039	1.013	1.006
CTSS	0.867	0.868	0.874	0.889	0.884	0.868
CXCR2	0.793	0.803	0.806	0.916	0.896	0.889
DAPK3	0.62	0.678	0.665	1.001	0.938	0.954
DRD1	0.812	0.81	0.819	0.877	0.882	0.862
DRD2	0.792	0.79	0.801	0.864	0.868	0.852
DRD3	0.837	0.837	0.845	0.880	0.881	0.861
DRD4	0.759	0.773	0.775	0.945	0.919	0.915
DRD5	0.851	0.848	0.857	0.910	0.918	0.894
ERBB4	0.772	0.788	0.792	1.014	0.985	0.979
ERN1	0.724	0.725	0.734	0.858	0.858	0.847
GCGR	0.822	0.818	0.829	0.749	0.756	0.735
GHSR	0.715	0.722	0.731	1.048	1.038	1.023
GLRA1	0.706	0.74	0.74	0.670	0.644	0.645
GRIK1	0.67	0.626	0.661	1.009	1.064	1.019
GRIN2B	0.636	0.628	0.646	1.011	1.020	1.001
GRM1	0.814	0.815	0.822	0.941	0.938	0.922
GRM2	0.846	0.85	0.855	0.651	0.642	0.634
GRM3	0.738	0.733	0.744	0.943	0.947	0.933
GRM4	0.823	0.816	0.825	1.340	1.409	1.362
GRM5	0.737	0.744	0.751	0.903	0.892	0.880
HAP1	0.668	0.698	0.698	0.710	0.685	0.687
HCAR2	0.802	0.792	0.802	0.830	0.851	0.830
HCRTR2	0.817	0.817	0.826	0.799	0.798	0.781
HDAC6	0.837	0.842	0.848	0.824	0.813	0.799

HIF1A	0.756	0.75	0.762	0.861	0.871	0.852
HSP90AA1	0.831	0.841	0.843	0.679	0.661	0.657
HTR1A	0.82	0.825	0.831	0.885	0.874	0.861
HTR1B	0.786	0.789	0.798	1.060	1.052	1.037
HTR1D	0.888	0.887	0.894	0.856	0.861	0.839
HTR1E	0.578	0.568	0.585	0.699	0.706	0.697
HTR2A	0.828	0.83	0.838	0.862	0.859	0.841
HTR2C	0.77	0.771	0.781	0.887	0.886	0.869
HTR4	0.856	0.864	0.866	0.954	0.928	0.921
HTR5A	0.777	0.791	0.79	0.957	0.932	0.934
HTR6	0.856	0.859	0.865	0.806	0.797	0.782
HTR7	0.797	0.792	0.804	0.845	0.854	0.834
KCNK3	0.618	0.63	0.639	0.711	0.699	0.690
KEAP1	0.747	0.71	0.753	1.047	1.109	1.040
LPAR1	0.749	0.749	0.757	0.864	0.862	0.851
LTB4R	0.761	0.754	0.766	1.029	1.040	1.019
MAOB	0.8	0.804	0.81	0.985	0.975	0.962
MCL1	0.871	0.864	0.874	0.672	0.690	0.665
MRGPRX4	0.535	0.485	0.525	0.624	0.654	0.630
MTNR1A	0.814	0.819	0.827	1.096	1.082	1.062
MTNR1B	0.788	0.804	0.806	1.095	1.058	1.053
MTOR	0.873	0.87	0.877	0.760	0.768	0.748
NLRP3	0.765	0.775	0.778	0.735	0.720	0.716
NOD2	0.727	0.725	0.733	0.809	0.810	0.800
NPY1R	0.851	0.824	0.855	0.960	1.035	0.949
NPY2R	0.881	0.846	0.883	0.927	1.048	0.931
NTRK2	0.844	0.844	0.849	1.028	1.028	1.011
NTSR1	0.774	0.748	0.779	1.422	1.498	1.406
OPRD1	0.856	0.846	0.86	0.951	0.981	0.938
OPRM1	0.836	0.83	0.842	0.968	0.983	0.951
OXTR	0.761	0.745	0.767	0.987	1.019	0.976
P2RY12	0.845	0.844	0.852	0.776	0.780	0.761
PIK3C3	0.53	0.481	0.519	1.128	1.167	1.137
PIK3CB	0.85	0.841	0.852	0.863	0.886	0.857
PIK3CD	0.828	0.829	0.835	0.905	0.903	0.888
PIK3CG	0.814	0.817	0.823	0.882	0.876	0.862
PKMYT1	0.708	0.718	0.718	1.033	1.017	1.017
PRKAA1	0.62	0.641	0.647	0.928	0.907	0.903
PRKCE	0.713	0.705	0.72	1.068	1.080	1.056
PRLHR	0.685	0.702	0.713	0.536	0.524	0.519
PTGER1	0.848	0.834	0.847	0.876	0.913	0.879
PTGER2	0.771	0.775	0.786	0.866	0.859	0.841
PTGER3	0.854	0.855	0.861	0.829	0.825	0.809
PTGER4	0.822	0.815	0.825	1.007	1.024	0.997
RIPK1	0.547	0.567	0.567	1.162	1.133	1.133
RIPK2	0.688	0.685	0.697	1.057	1.061	1.047
RPS6KB1	0.863	0.861	0.868	0.841	0.847	0.827
S1PR1	0.859	0.856	0.864	1.015	1.023	0.997
S1PR2	0.844	0.847	0.854	0.783	0.776	0.762

S1PR3	0.788	0.794	0.802	0.936	0.923	0.908
S1PR4	0.799	0.793	0.803	0.679	0.688	0.672
SCN10A	0.837	0.828	0.839	0.906	0.928	0.903
SCN4A	0.658	0.552	0.684	0.962	1.065	0.959
SCN5A	0.71	0.69	0.712	0.719	0.740	0.715
SCN9A	0.84	0.824	0.843	0.732	0.763	0.725
SIRT1	0.798	0.81	0.813	0.801	0.779	0.774
SIRT3	0.782	0.797	0.799	0.868	0.841	0.840
SLC1A1	0.636	0.651	0.652	0.922	0.906	0.904
SLC1A2	0.83	0.808	0.826	0.787	0.830	0.797
SLC1A3	0.701	0.673	0.696	0.961	0.996	0.967
SLC6A1	0.671	0.662	0.679	1.007	1.020	0.997
SLC6A3	0.83	0.83	0.838	0.845	0.844	0.828
SLC6A4	0.82	0.823	0.83	0.891	0.885	0.868
SLC6A5	0.82	0.814	0.825	0.858	0.870	0.846
SSTR1	0.795	0.774	0.804	0.889	0.923	0.866
SSTR2	0.845	0.836	0.854	1.028	1.055	1.004
SSTR3	0.768	0.73	0.766	0.909	0.973	0.909
SSTR4	0.881	0.855	0.882	0.835	0.917	0.835
SSTR5	0.853	0.842	0.862	0.883	0.911	0.860
STK3	0.655	0.632	0.656	0.820	0.841	0.819
TAS2R8	0.766	0.769	0.777	0.597	0.593	0.584
TBK1	0.712	0.721	0.727	1.009	0.995	0.985
USP30	0.642	0.639	0.652	0.644	0.647	0.636
VCP	0.746	0.771	0.769	0.771	0.739	0.741
hERG	0.764	0.758	0.773	0.773	0.782	0.765
Average	0.779	0.775	0.788	0.900	0.905	0.883

Table S5: The performance comparisons of the transformer, autoencoder fingerprints and their consensus in the ten-fold cross validation tests.

Abbreviations	Full name
AAK1	AP2-associated protein kinase 1
ADORA1	Adenosine receptor A1
ADORA3	Adenosine receptor A3
ADRA2A	Alpha-2A adrenergic receptor
ADRB1	Beta-1 adrenergic receptor
ADRB2	Beta-2 adrenergic receptor
ADRB3	Beta-3 adrenergic receptor
AGTR2	Type-2 angiotensin II receptor
AKT1	RAC-alpha serine/threonine-protein kinase
APLNR	Apelin receptor
ATG4B	Cysteine protease ATG4B
ATG7	Ubiquitin-like modifier-activating enzyme ATG7
BCL2L1	Bcl-2-like protein 1
BDKRB2	B2 bradykinin receptor
CACNA1B	Voltage-dependent N-type calcium channel subunit alpha-1B
CACNA1C	Voltage-dependent L-type calcium channel subunit alpha-1C
CACNA1G	Voltage-dependent T-type calcium channel subunit alpha-1G

CACNA1H	Voltage-dependent T-type calcium channel subunit alpha-1H
CACNA1I	Voltage-dependent T-type calcium channel subunit alpha-1I
CALCR	Calcitonin receptor
CALCRL	Calcitonin gene-related peptide type 1 receptor
CASP3	Caspase-3
CASP8	Caspase-8
CCKAR	Cholecystokinin receptor type A
CCR3	C-C chemokine receptor type 3
CCR4	C-C chemokine receptor type 4
CHRM1	Muscarinic acetylcholine receptor M1
CHRM2	Muscarinic acetylcholine receptor M2
CHRM3	Muscarinic acetylcholine receptor M3
CHRM4	Muscarinic acetylcholine receptor M4
CHRNA7	Neuronal acetylcholine receptor subunit alpha-7
CNR1	Cannabinoid receptor 1
CNR2	Cannabinoid receptor 2
CTSB	Cathepsin B
CTSD	Cathepsin D
CTSL	Cathepsin L
CTSS	Cathepsin S
CXCR2	C-X-C chemokine receptor type 2
DAPK3	Death-associated protein kinase 3
DRD1	D(1A) dopamine receptor
DRD2	D(2) dopamine receptor
DRD3	D(3) dopamine receptor
DRD4	D(4) dopamine receptor
DRD5	D(1B) dopamine receptor
ERBB4	Receptor tyrosine-protein kinase erbB-4
ERN1	Serine/threonine-protein kinase/endoribonuclease IRE1
GABRA5	Gamma-aminobutyric acid receptor subunit alpha-5
GCGR	Glucagon receptor
GHSR	Growth hormone secretagogue receptor type 1
GLRA1	Glycine receptor subunit alpha-1
GRIK1	Serine/threonine-protein kinase GRIK1
GRIN2B	Glutamate receptor ionotropic, NMDA 2B
GRM1	Metabotropic glutamate receptor 1
GRM2	Metabotropic glutamate receptor 2
GRM3	Metabotropic glutamate receptor 3
GRM4	Metabotropic glutamate receptor 4
GRM5	Metabotropic glutamate receptor 5
HAP1	Huntingtin-associated protein 1
HCAR2	Hydroxycarboxylic acid receptor 2
HCRTR2	Orexin receptor type 2
HDAC6	Histone deacetylase 6
hERG	hERG Potassium Channel
HIF1A	Hypoxia-inducible factor 1-alpha
HSP90AA1	Heat shock protein HSP 90-alpha
HTR1A	5-hydroxytryptamine receptor 1A
HTR1B	5-hydroxytryptamine receptor 1B

HTR1D	5-hydroxytryptamine receptor 1D
HTR1E	5-hydroxytryptamine receptor 1E
HTR2A	5-hydroxytryptamine receptor 2A
HTR2C	5-hydroxytryptamine receptor 2C
HTR4	5-hydroxytryptamine receptor 4
HTR5A	5-hydroxytryptamine receptor 5A
HTR6	5-hydroxytryptamine receptor 6
HTR7	5-hydroxytryptamine receptor 7
KCNK3	Potassium channel subfamily K member 3
KEAP1	Kelch-like ECH-associated protein 1
LPAR1	Lysophosphatidic acid receptor 1
LTB4R	Leukotriene B4 receptor 1
MAOB	Amine oxidase [flavin-containing] B
MCL1	Induced myeloid leukemia cell differentiation protein Mcl-1
MRGPRX4	Mas-related G-protein coupled receptor member X4
MTNR1A	Melatonin receptor type 1A
MTNR1B	Melatonin receptor type 1B
MTOR	Serine/threonine-protein kinase mTOR
NLRP3	NACHT, LRR and PYD domains-containing protein 3
NOD2	Nucleotide-binding oligomerization domain-containing protein 2
NPY1R	Neuropeptide Y receptor type 1
NPY2R	Neuropeptide Y receptor type 2
NTRK2	BDNF/NT-3 growth factors receptor
NTSR1	Neurotensin receptor type 1
OPRD1	Delta-type opioid receptor
OPRM1	Mu-type opioid receptor
OXTR	Oxytocin receptor
P2RY12	P2Y purinoceptor 12
PIK3C3	Phosphatidylinositol 3-kinase catalytic subunit type 3
PIK3CB	Phosphatidylinositol 4,5-bisphosphate 3-kinase catalytic subunit beta isoform
PIK3CD	Phosphatidylinositol 4,5-bisphosphate 3-kinase catalytic subunit delta isoform
PIK3CG	Phosphatidylinositol 4,5-bisphosphate 3-kinase catalytic subunit gamma isoform
PKMYT1	Membrane-associated tyrosine- and threonine-specific cdc2-inhibitory kinase
PRKAA1	5'-AMP-activated protein kinase catalytic subunit alpha-1
PRKCE	Protein kinase C epsilon type
PRLHR	Prolactin-releasing peptide receptor
PTGER1	Prostaglandin E2 receptor EP1 subtype
PTGER2	Prostaglandin E2 receptor EP2 subtype
PTGER3	Prostaglandin E2 receptor EP3 subtype
PTGER4	Prostaglandin E2 receptor EP4 subtype
RIPK1	Receptor-interacting serine/threonine-protein kinase 1
RIPK2	Receptor-interacting serine/threonine-protein kinase 2
RPS6KB1	Ribosomal protein S6 kinase beta-1
S1PR1	Sphingosine 1-phosphate receptor 1
S1PR2	Sphingosine 1-phosphate receptor 2
S1PR3	Sphingosine 1-phosphate receptor 3
S1PR4	Sphingosine 1-phosphate receptor 4
SCN10A	Sodium channel protein type 10 subunit alpha
SCN4A	Sodium channel protein type 4 subunit alpha

SCN5A	Sodium channel protein type 5 subunit alpha
SCN9A	Sodium channel protein type 9 subunit alpha
SIRT1	NAD-dependent protein deacetylase sirtuin-1
SIRT3	NAD-dependent protein deacetylase sirtuin-3, mitochondrial
SLC1A1	Excitatory amino acid transporter 3
SLC1A2	Excitatory amino acid transporter 2
SLC1A3	Excitatory amino acid transporter 1
SLC6A1	Sodium- and chloride-dependent GABA transporter 1
SLC6A3	Sodium-dependent dopamine transporter
SLC6A4	Sodium-dependent serotonin transporter
SLC6A5	Sodium- and chloride-dependent glycine transporter 2
SSTR1	Somatostatin receptor type 1
SSTR2	Somatostatin receptor type 2
SSTR3	Somatostatin receptor type 3
SSTR4	Somatostatin receptor type 4
SSTR5	Somatostatin receptor type 5
STK3	Serine/threonine-protein kinase 3
TAS2R8	Taste receptor type 2 member 8
TBK1	Serine/threonine-protein kinase TBK1
USP30	Ubiquitin carboxyl-terminal hydrolase 30
VCP	Transitional endoplasmic reticulum ATPase

Table S6: The abbreviations of protein names and the corresponding full protein names.

S1.3 Protein-protein interactions in the core networks

We showed three PPI networks (GABRA1, GABRA5, and GABRR3) in Table S7. Within each network, there is a core subnetwork whose proteins interact directly with specific GABA receptors. The second and third columns of Table S7 records the abbreviations and full names of the proteins, respectively. Also, there are 11 proteins in each network.

Regression		Metrics					
		Average R			Average RMSD		
Dataset	fingerprint	GBDT	SVM	RF	GBDT	SVM	RF
GABRA5 (381)	BET	0.721	0.778	0.66	0.724	0.648	0.781
	AE	0.737	0.781	0.692	0.714	0.644	0.756
	consensus (BET+AE)	0.741	0.788	0.691	0.710	0.634	0.759
hERG (7320)	BET	0.767	0.764	0.623	0.773	0.773	0.960
	AE	0.755	0.758	0.641	0.802	0.782	0.947
	consensus (BET+AE)	0.778	0.773	0.672	0.770	0.765	0.939
ADORA1 (3611)	BET	0.753	0.777	0.685	0.930	0.875	1.085
	AE	0.75	0.781	0.654	0.940	0.868	1.099
	consensus (BET+AE)	0.764	0.789	0.686	0.921	0.854	1.084
ADORA3 (3403)	BET	0.837	0.853	0.761	0.955	0.888	1.159
	AE	0.832	0.851	0.767	0.991	0.893	1.149
	consensus (BET+AE)	0.843	0.86	0.783	0.956	0.870	1.135
BCL2L1 (855)	BET	0.886	0.902	0.858	0.943	0.853	1.050
	AE	0.886	0.901	0.867	0.975	0.856	0.995
	consensus (BET+AE)	0.895	0.909	0.876	0.932	0.827	0.988
CASP3 (3911)	BET	0.81	0.836	0.732	1.049	0.968	1.234
	AE	0.808	0.83	0.745	1.071	0.983	1.218
	consensus (BET+AE)	0.818	0.842	0.754	1.043	0.951	1.210
CHRM3 (1677)	BET	0.874	0.892	0.843	1.097	1.004	1.231
	AE	0.873	0.898	0.791	1.178	0.980	1.431
	consensus (BET+AE)	0.882	0.9	0.845	1.107	0.971	1.280
SLC6A5 (2526)	BET	0.792	0.82	0.719	0.930	0.858	1.077
	AE	0.786	0.814	0.732	0.959	0.870	1.074
	consensus (BET+AE)	0.799	0.825	0.745	0.931	0.846	1.060

Table S4: Comparisons of GBDT, SVM and RF models in the ten-fold cross-validation tests. The numbers inside the parenthesis indicate the size of the training set.

Network	Abbreviations	Protein full names
Core network of GABRA1	GABRA1	Gamma-aminobutyric acid receptor subunit alpha-1
	CLCN2	Chloride channel protein 2
	GABBR2	Gamma-aminobutyric acid type B receptor subunit 2
	GABRB1	Gamma-aminobutyric acid receptor subunit beta-1
	GABRB2	Gamma-aminobutyric acid receptor subunit beta-2
	GABRB3	Gamma-aminobutyric acid receptor subunit beta-3
	GABRG2	Gamma-aminobutyric acid receptor subunit gamma-2
	HAP1	Huntingtin-associated protein 1
	NSF	Vesicle-fusing ATPase
	PLCL1	Inactive phospholipase C-like protein 1
	TRAK2	Trafficking kinesin-binding protein 2
Core network of GABRA5	GABRA5	Gamma-aminobutyric acid receptor subunit alpha-5
	GABBR1	Gamma-aminobutyric acid type B receptor subunit 1
	GABBR2	Gamma-aminobutyric acid type B receptor subunit 2
	GABRA3	Gamma-aminobutyric acid receptor subunit alpha-3
	GABRB1	Gamma-aminobutyric acid receptor subunit beta-1
	HAP1	Huntingtin-associated protein 1
	NSF	Vesicle-fusing ATPase
	PLCL1	Inactive phospholipase C-like protein 1
	SLC6A1	Sodium- and chloride-dependent GABA transporter 1
	SLC6A13	Sodium- and chloride-dependent GABA transporter 2
	TRAK2	Trafficking kinesin-binding protein 2
Core network of GABRR3	GABRR3	Gamma-aminobutyric acid receptor subunit rho-3
	GABBR2	Gamma-aminobutyric acid type B receptor subunit 2
	GABRA5	Gamma-aminobutyric acid receptor subunit alpha-5
	GABRB1	Gamma-aminobutyric acid receptor subunit beta-1
	GABRB2	Gamma-aminobutyric acid receptor subunit beta-2
	GABRE	Gamma-aminobutyric acid receptor subunit epsilon
	GABRG1	Gamma-aminobutyric acid receptor subunit gamma-1
	GABRG2	Gamma-aminobutyric acid receptor subunit gamma-2
	GABRQ	Gamma-aminobutyric acid receptor subunit theta
	GABRR1	Gamma-aminobutyric acid receptor subunit rho-1
	GABRR2	Gamma-aminobutyric acid receptor subunit rho-2

Table S7: The abbreviations and full names of the proteins in the three core PPI networks of GABA receptors.

S2 More ADMET properties and optimal ranges

In the present work, we have selected five ADMET properties to find the near-optimal compound lead compounds. ADMET includes multiple properties for pharmacokinetic studies of compounds. By repurposing them, we obtained 2 near-optimal lead compounds. We further evaluated some other AMDET properties of these two compounds as shown in Figure 6 in the main text. All these properties are described in Table S8.

Property Names	Formulations of properties	Optimal range
MW	Molecular Weight, contains hydrogen atoms	100-600
Volume	Van der Waals volumn	-
Density	MW/Volumn	-
nHA	Number of hydrogen bond acceptors	0-12
nRot	Number of rotatable bonds	0-11
nRing	Number of rings	0-6
MaxRing	Number of atoms in the biggest ring	0-18
nHet	Number of heteroatoms	1-15
fChar	Formal charge	-4-4
nRig	Number of rigid bonds	0-30
Flexibility	nRot/ nRig	-
Stereo Centers		≤ 2
TPSA	Topological polar surface area	0-140
Log S	log of the aqueous solubility	-4-0.5 log mol/L
Log P	log of octanol/water partition coefficient	0-3
Log D	log P at physiological pH 7.4	1-3

Table S8: The optimal ranges of 13 selected ADMET characteristics.

S3 Additional results of predicted correlations between BA and similar proteins

SSTR1, SSTR2, SSTR3, SSTR4, and SSTR5 belong to the G-protein-coupled receptor (GPCR) family, which have highly similar 3D structural conformations as shown in Figure S2. The high degree of similarity in the 2D sequences underlies their structural similarity.

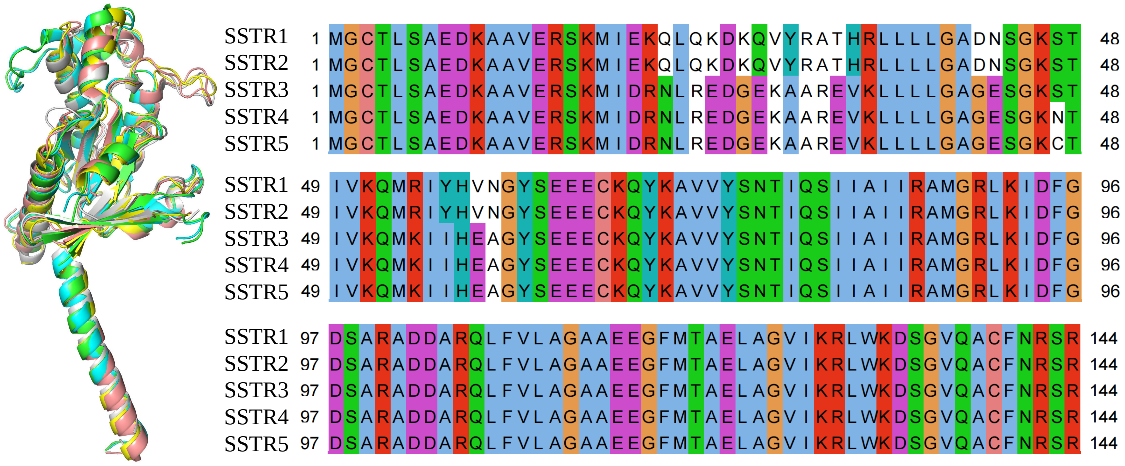

Figure S2: The alignment of 3D structures and 2D sequences of the five proteins. The PDB IDs are 8XIO, 7WIC [1], 8XIR, 7XMT [14] and 8ZCJ [7] for SSTR1, SSTR2, SSTR3, SSTR4 and SSTR5, respectively.

The Pearson correlation coefficient is a statistic that measures the degree of linear correlation between two variables. It indicates the strength and direction of the linear relationship between two variables and

can take values between -1 and 1. The Pearson correlation coefficient can be calculated using the following formula:

$$R = \frac{\sum(x_i - \bar{\mathbf{x}})(y_i - \bar{\mathbf{y}})}{\sqrt{\sum(x_i - \bar{\mathbf{x}})^2 \sum(y_i - \bar{\mathbf{y}})^2}}, \tag{1}$$

where x_i and y_i denote the observed values of the two variables, and $\bar{x}$ and $\bar{y}$ denote the mean values of the two variables, respectively.

S4 Side effect predictions of existing medications

Cinchocaine, also known as dibucaine, is a potent local anesthetic belonging to the amide class. It is widely recognized for its long-lasting anesthetic effects and is often used in procedures requiring prolonged numbness, such as spinal and epidural anesthesia. Cinchocaine works by inhibiting sodium ion influx through nerve cell membranes, effectively blocking nerve signal transmission and preventing the sensation of pain. Due to its high potency and extended duration of action, cinchocaine is particularly useful in clinical settings where sustained anesthesia is essential. Additionally, it is commonly incorporated into topical formulations for the relief of pain and itching associated with hemorrhoids and other anorectal disorders [3]. Our model predicted the BA of cinchocaine to GABRA5 as -10.25 kcal/mol and to hERG as -8.35 kcal/mol. The latter value is below the hERG side effect threshold of -8.18 kcal/mol, suggesting that cinchocaine may interact with the hERG channel. This interaction indicates a potential risk of hERG-related side effects, such as arrhythmia. Furthermore, the most significant predicted BAs are for CALCR, CCKAR, and HTR4, with values of -12.78, -11.09, and -10.92 kcal/mol, respectively. These predictions align with the fact that cinchocaine is a local anesthetic with potential cardiac risks.

Remifentanil, alfentanil, and fentanyl are three intravenous opioids commonly used in clinical practice as general anesthetics [10]. Remifentanil is an ultra-short-acting opioid with a rapid metabolic rate, primarily utilized for anesthetic procedures requiring quick onset and offset. In contrast, alfentanil, a short-acting opioid, has a faster onset than fentanyl and is frequently used for short surgical procedures and analgesia. Fentanyl, a potent opioid approximately 100 times stronger than morphine, is commonly employed for intra-operative analgesia, postoperative pain management, and chronic pain treatment. Our predictions indicate that the BAs of remifentanil, alfentanil, and fentanyl for GABRA5 are -10.42, -10.81, and -10.55 kcal/mol, respectively, while their BAs for hERG are -7.64, -7.58, and -8.86 kcal/mol, respectively. Consequently, remifentanil and alfentanil exhibit no adverse effects on hERG, and indeed, they are approved by the FDA. On the other hand, fentanyl shows a potential risk to hERG, which could affect cardiac electrophysiology, consistent with the findings of Sherbini et al. [4]. Further computational analysis reveals that the protein with the highest BA for these three medications is CALCR, with BAs of -12.92, -12.88, and -12.66 kcal/mol, respectively. This high affinity suggests potential alterations in calcium metabolism, bone health, or other related physiological functions, implying the complex pharmacological characteristics of these anesthetics with multiple targets. Thus, the high BA to CALCR should be considered when using these drugs due to the additional implications it may have.

Sevoflurane, desflurane, and isoflurane are three commonly used inhalation anesthetics that act as positive allosteric modulators of GABA receptors, widely employed in surgical procedures to induce and maintain general anesthesia [11]. Sevoflurane is characterized by its rapid onset and recovery, as well as its relatively mild odor, making it frequently used in pediatric anesthesia. Desflurane, with the lowest blood-gas partition coefficient, provides the fastest induction and recovery times; however, its high volatility requires a specialized vaporizer and can cause airway irritation. Isoflurane is renowned for its stable hemodynamic properties and minimal metabolic byproducts, making it suitable for patients undergoing cardiac surgery. Our model predicts the (BAs of sevoflurane, desflurane, and isoflurane to GABRA5 to be -11.04, -11.40, and -11.59 kcal/mol, respectively. This confirms that these anesthetics exert their effects by enhancing GABRA5 activity, promoting chloride channel opening, and inhibiting neuronal hyperexcitability. Furthermore, their

BAs to hERG are -7.49, -7.36, and -7.68 kcal/mol, respectively, validating that these anesthetics do not have adverse effects on hERG. Additionally, these three anesthetics exhibit very high BAs to both CALCR and MTNR1B, with BAs of -12.82, -12.92, and -13.08 kcal/mol for CALCR and -11.45, -11.49, and -11.51 kcal/mol for MTNR1B, respectively. Thus, these inhalation anesthetics may have potential effects on CALCR and MTNR1B.

Propofol, midazolam, diazepam, lorazepam, and etomidate are all positive allosteric modulators of the GABA receptor, administered intravenously (non-opioid). They primarily target the $\mathrm{GABA_A}$ receptor, enhancing the inhibitory neurotransmission of GABA, thereby providing sedative, anxiolytic, anticonvulsant, and anesthetic effects [8].

Propofol is a rapid-acting intravenous anesthetic commonly used for the induction and maintenance of general anesthesia, as well as for sedation in intensive care units. It is characterized by its quick onset and short duration of action, although it may cause hypotension and respiratory depression. Midazolam, a benzodiazepine, offers sedative, anxiolytic, and anticonvulsant properties and is frequently utilized for preoperative sedation and sedation during endoscopic procedures. Its advantages include rapid onset and short duration of action.

Diazepam, another benzodiazepine, is widely used for treating anxiety disorders, relieving muscle spasms, and controlling status epilepticus, and it has a longer duration of action compared to midazolam. Lorazepam, also a benzodiazepine, is primarily used for short-term treatment of anxiety disorders and insomnia, as well as for preoperative sedation, offering a longer duration of action and fewer drug interactions.

Etomidate is a short-acting intravenous anesthetic commonly used for the induction of general anesthesia, particularly suitable for patients with unstable cardiovascular function due to its minimal effects on the cardiovascular system. However, etomidate may cause adrenal cortical suppression.

Our model predicts the BAs of propofol, midazolam, diazepam, lorazepam, and etomidate to GABRA5 as -10.33, -10.69, -10.52, -10.60, and -11.05 kcal/mol, respectively. Additionally, their BAs to hERG are -7.92, -7.48, -7.86, -8.13, and -7.29 kcal/mol, respectively, confirming that these FDA-approved anesthetics lack significant hERG-related side effects. Furthermore, these five anesthetics exhibit the highest BAs with CALCR, specifically -13.16, -12.54, -12.53, -12.93, and -12.82 kcal/mol, suggesting a higher potential for CALCR-related side effects.

We used our model to assess the potential side effects of existing anesthetics in the PPI network. Figure S9 provides the 2D structure of an existing anesthetic. The predicted BA values for several anesthetics with potential side effects are shown next to their 2D structures. By looking at these BA values, we are able to find out the effect and side effect profiles of these anesthetics.

S5 Repurposing potentials to GABRA5 and side effects on hERG

The x-axis of each panel in Figures S10, S11, S12, and S13 shows the utilization potential of the 134 inhibitor datasets for GABRA5. 134 side effects of the group of hERG inhibitors are also predicted and shown in the y-axis of each panel. The orange frame outlined in these figures allow us to estimate the number of compounds in each dataset that have the repurposing potential for the specified target and have no side effects on hERG.

S6 The hERG screening

The hERG protein encodes a voltage-gated potassium channel, also known as Kv11.1 or the hERG channel [12, 13]. Many drugs can inadvertently inhibit the hERG channel, reducing the IKr current and thereby prolonging the repolarization time of the cardiac action potential (QT interval prolongation) [6, 9]. QT

interval prolongation can lead to fatal arrhythmias, such as torsades de pointes. Therefore, it is essential to test new drugs for hERG channel inhibition during drug development to ensure their safety [2, 13]. We constructed a machine learning model based on seven datasets as described in [5]. Our model was validated and compared favorably with many other state-of-art methods. In our approach, we set a stringent side effect threshold of -8.18 kcal/mol (K_i=1 μM). If a compound's binding affinity value with the hERG channel is smaller than -8.18 kcal/mol (i.e., more negative), it is denoted as unsafe in clinical application.

S7 ADMET properties of existing drugs

We also collected ADMET property maps for the 15 drugs in Figure S9 and all the images are presented in Figure S14, which are available at https://admetmesh.scbdd.com/. By looking at these pictures, for some of the properties that do not meet the requirements, we can perform molecular optimization to get compounds with better properties.

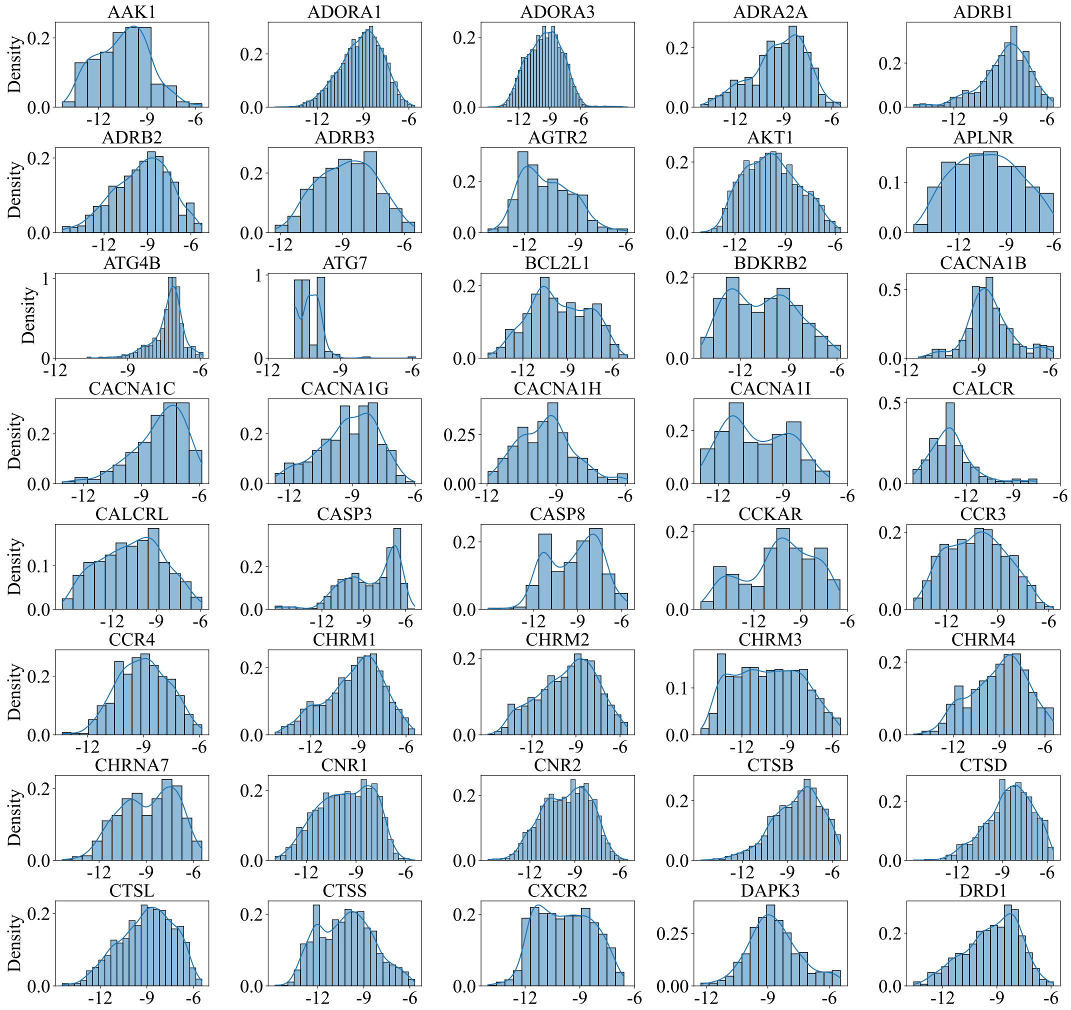

Figure S3: Label distribution of the inhibitor dataset for the top 40 protein targets in Table S1.

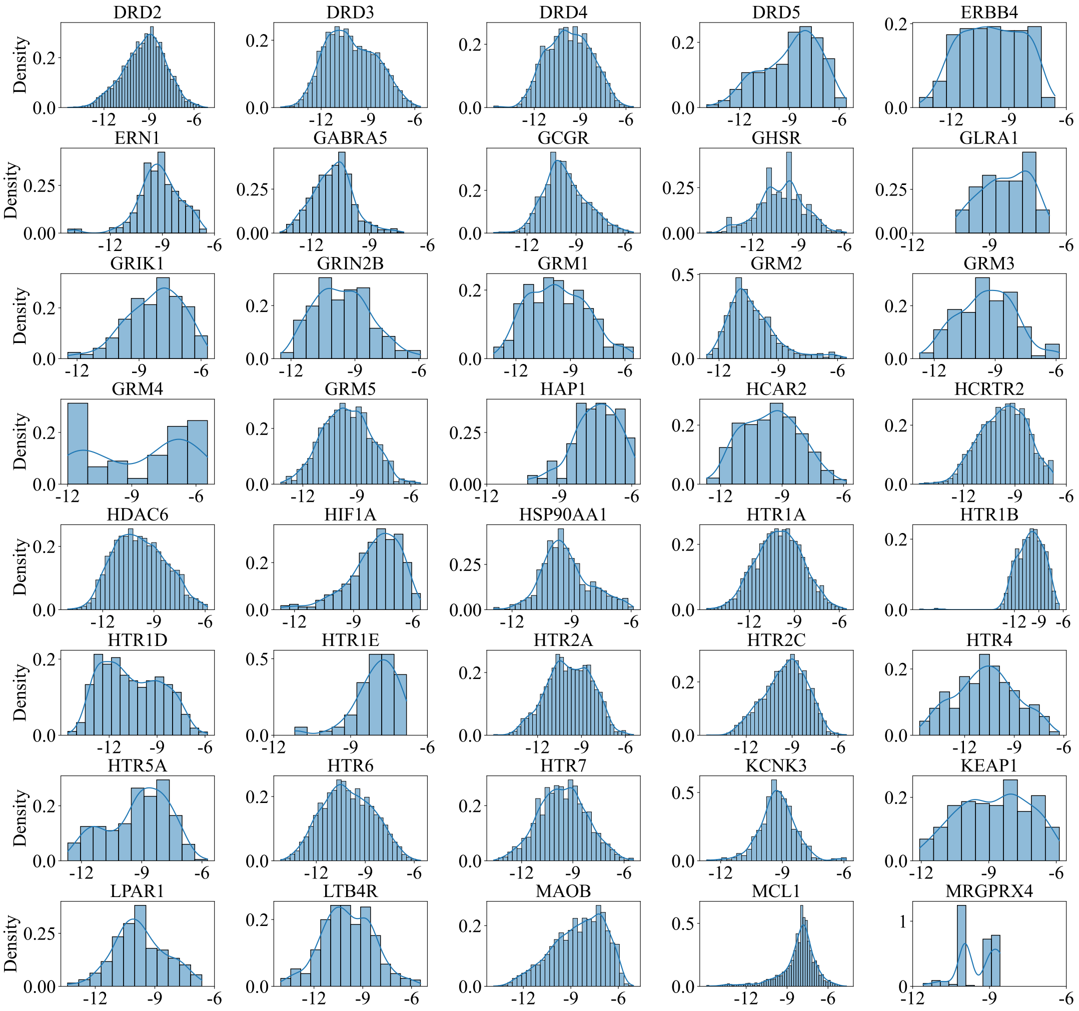

Figure S4: Label distribution of the inhibitor dataset for the next 40 protein targets in Table S1.

mode	affinity (kcal/mol)	dist from rmsd l.b.	best mode rmsd u.b.
1	-7.3	0.000	0
2	-7.2	18.166	19.672
3	-7.2	2.624	6.554
4	-7.0	1.864	3.193
5	-6.9	3.087	6.302
6	-6.9	17.361	19.501
7	-6.8	19.025	21.210
8	-6.7	18.078	19.464
9	-6.6	17.793	19.952

Table S9: Details of the docking between ChEMBL1372447 and GABRA5.

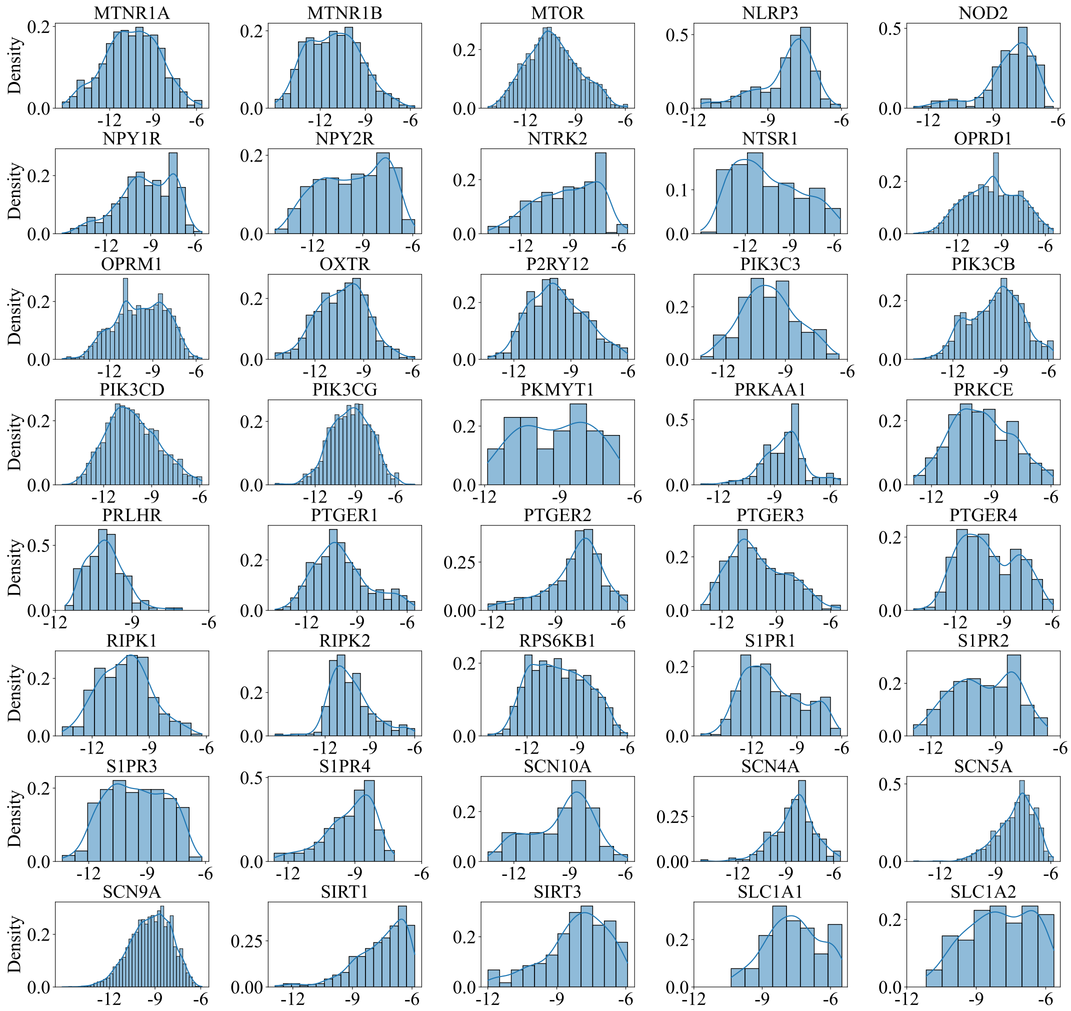

Figure S5: Label distribution of the inhibitor dataset for the next 40 protein targets in Table S1.

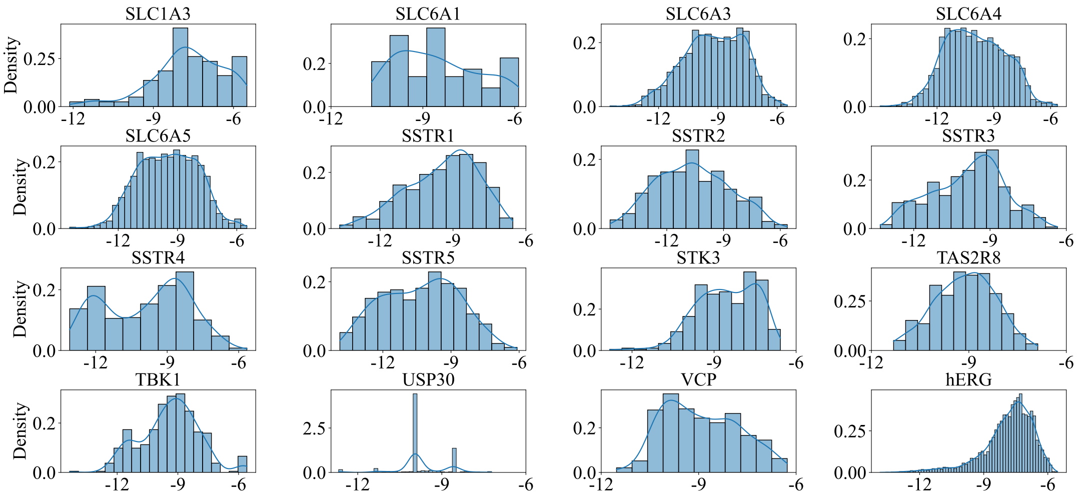

Figure S6: Label distribution of the inhibitor dataset for the last 16 protein targets in Table S1.

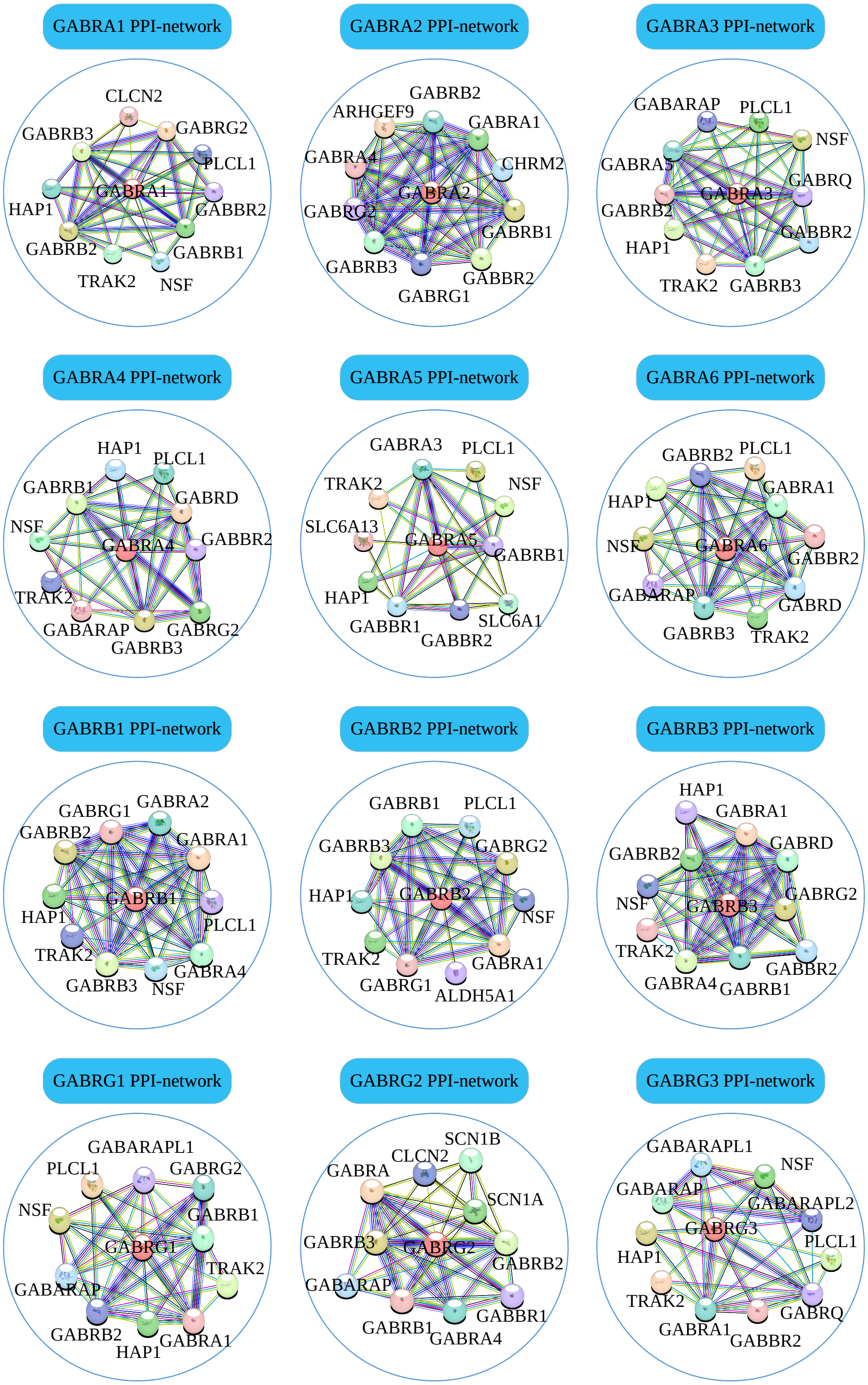

Figure S7: Protein-protein interactions in the first 12 core networks of GABA receptors.

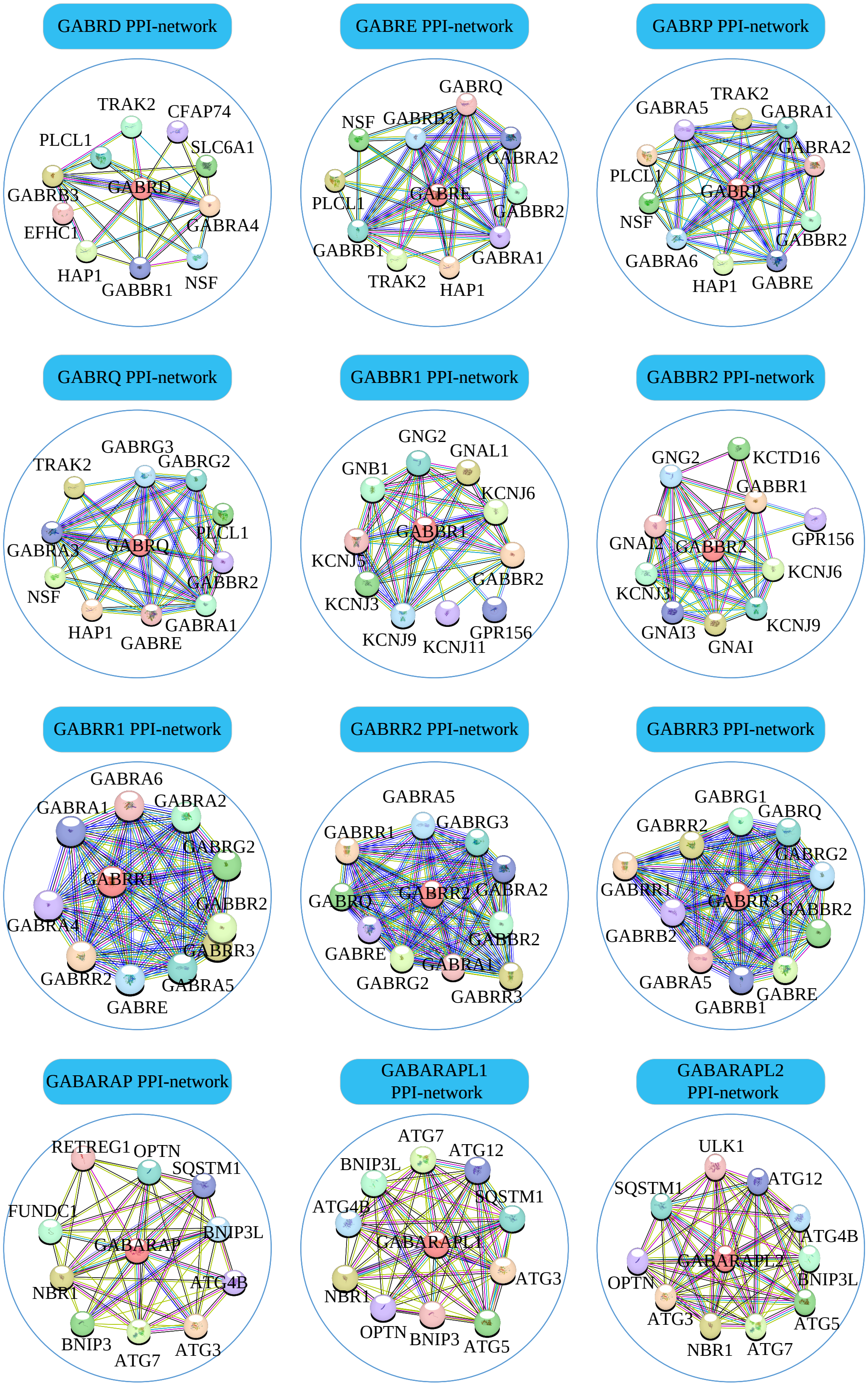

Figure S8: Protein-protein interactions in the last 12 core networks of GABA receptors.

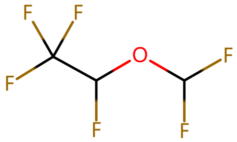

Desflurane
Predicted BA:
GABRA5: -11.40 kcal/mol
CALCR: -12.92 kcal/mol
MTNR1B: -11.49 kcal/mol
CCKAR: -11.19 kcal/mol
HTR4: -11.15 kcal/mol
hERG: -7.36 kcal/mol

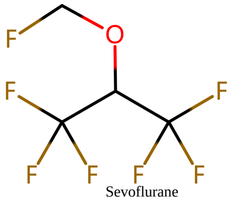

Sevoflurane
Predicted BA:
GABRA5: -11.04 kcal/mol
CALCR: -12.82 kcal/mol
MTNR1B: -11.45 kcal/mol
NTSR1: -10.96 kcal/mol
CCKAR: -10.74 kcal/mol
hERG: -7.49 kcal/mol

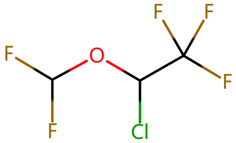

Isoflurane
Predicted BA:
GABRA5: -11.59 kcal/mol
CALCR: -13.08 kcal/mol
MTNR1B: -11.51 kcal/mol
CCKAR: -11.02 kcal/mol
HTR4: -10.97 kcal/mol
hERG: -7.68 kcal/mol

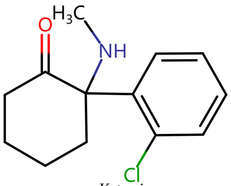

Ketamine
Predicted BA:
GABRA5: -10.40 kcal/mol
CALCR: -12.91 kcal/mol
MTNR1B: -10.89 kcal/mol
APLNR: -10.88 kcal/mol
CALCRL: -10.75 kcal/mol
hERG: -7.15 kcal/mol

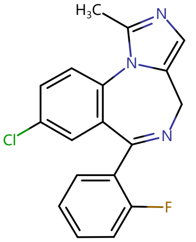

Midazolam
Predicted BA:
GABRA5: -10.69 kcal/mol
CALCR: -12.54 kcal/mol
PIK3CD: -11.23 kcal/mol
PIK3CG: -10.95 kcal/mol
S1PR1: -10.83 kcal/mol
hERG: -7.48 kcal/mol

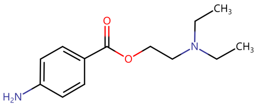

Procaine
Predicted BA:
GABRA5: -10.71 kcal/mol
CALCR: -12.77 kcal/mol
NTSR1: -10.85 kcal/mol
APLNR: -10.63 kcal/mol
SSTR2: -10.61 kcal/mol
hERG: -7.34 kcal/mol

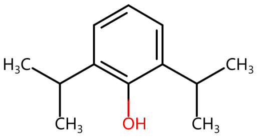

Propofol
Predicted BA:
GABRA5: -10.33 kcal/mol
CALCR: -13.16 kcal/mol
HTR1D: -11.47 kcal/mol
HTR1B: -10.92 kcal/mol
APLNR: -10.86 kcal/mol
hERG: -7.92 kcal/mol

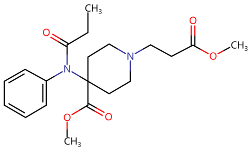

Remifentanil
Predicted BA:
GABRA5: -10.42 kcal/mol
CALCR: -12.92 kcal/mol
APLNR: -11.27 kcal/mol
CASP3: -11.09 kcal/mol
SSTR2: -10.80 kcal/mol
hERG: -7.64 kcal/mol

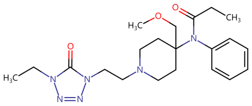

Alfentanil
Predicted BA:
GABRA5: -10.81 kcal/mol
CALCR: -12.88 kcal/mol
APLNR: -11.7 kcal/mol
HTR4: -11.59 kcal/mol
HTR1D: -11.49 kcal/mol
hERG: -7.58 kcal/mol

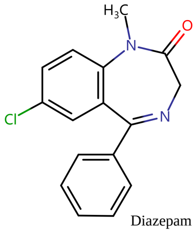

Diazepam
Predicted BA:
GABRA5: -10.52 kcal/mol
CALCR: -12.53 kcal/mol
MTNR1B: -12.01 kcal/mol
MTNR1A: -10.97 kcal/mol
NTSR1: -10.66 kcal/mol
hERG: -7.86 kcal/mol

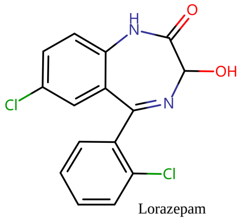

Lorazepam
Predicted BA:
GABRA5: -10.60 kcal/mol
CALCR: -12.93 kcal/mol
MTNR1B: -11.79 kcal/mol
OXTR: -10.94 kcal/mol
CALCRL: -10.78 kcal/mol
hERG: -8.13 kcal/mol

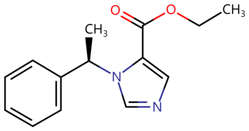

Etomidate
Predicted BA:
GABRA5: -11.05 kcal/mol
CALCR: -12.82 kcal/mol
APLNR: -10.64 kcal/mol
CALCRL: -10.53 kcal/mol
RIPK1: -10.52 kcal/mol
hERG: -7.29 kcal/mol

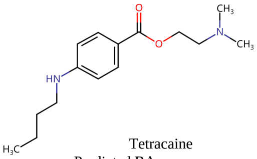

Tetracaine
Predicted BA:
GABRA5: -10.50 kcal/mol
CALCR: -13.37 kcal/mol
S1PR1: -10.81 kcal/mol
NTSR1: -10.79 kcal/mol
CCKAR: -10.62 kcal/mol
hERG: -8.02 kcal/mol

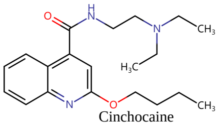

Cinchocaine
Predicted BA:
GABRA5: -10.25 kcal/mol
CALCR: -12.78 kcal/mol
CCKAR: -11.09 kcal/mol
HTR4: -10.92 kcal/mol
NPY2R: -10.8 kcal/mol
hERG: -8.35 kcal/mol

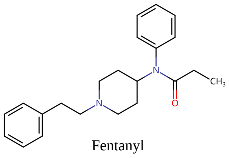

Fentanyl
Predicted BA:
GABRA5: -10.55 kcal/mol
CALCR: -12.66 kcal/mol
HTR4: -11.60 kcal/mol
HTR1D: -11.45 kcal/mol
SSTR2: -11.04 kcal/mol
hERG: -8.86 kcal/mol

Figure S9: The side effect predictions of the existing medications with the predicted binding energies shown.

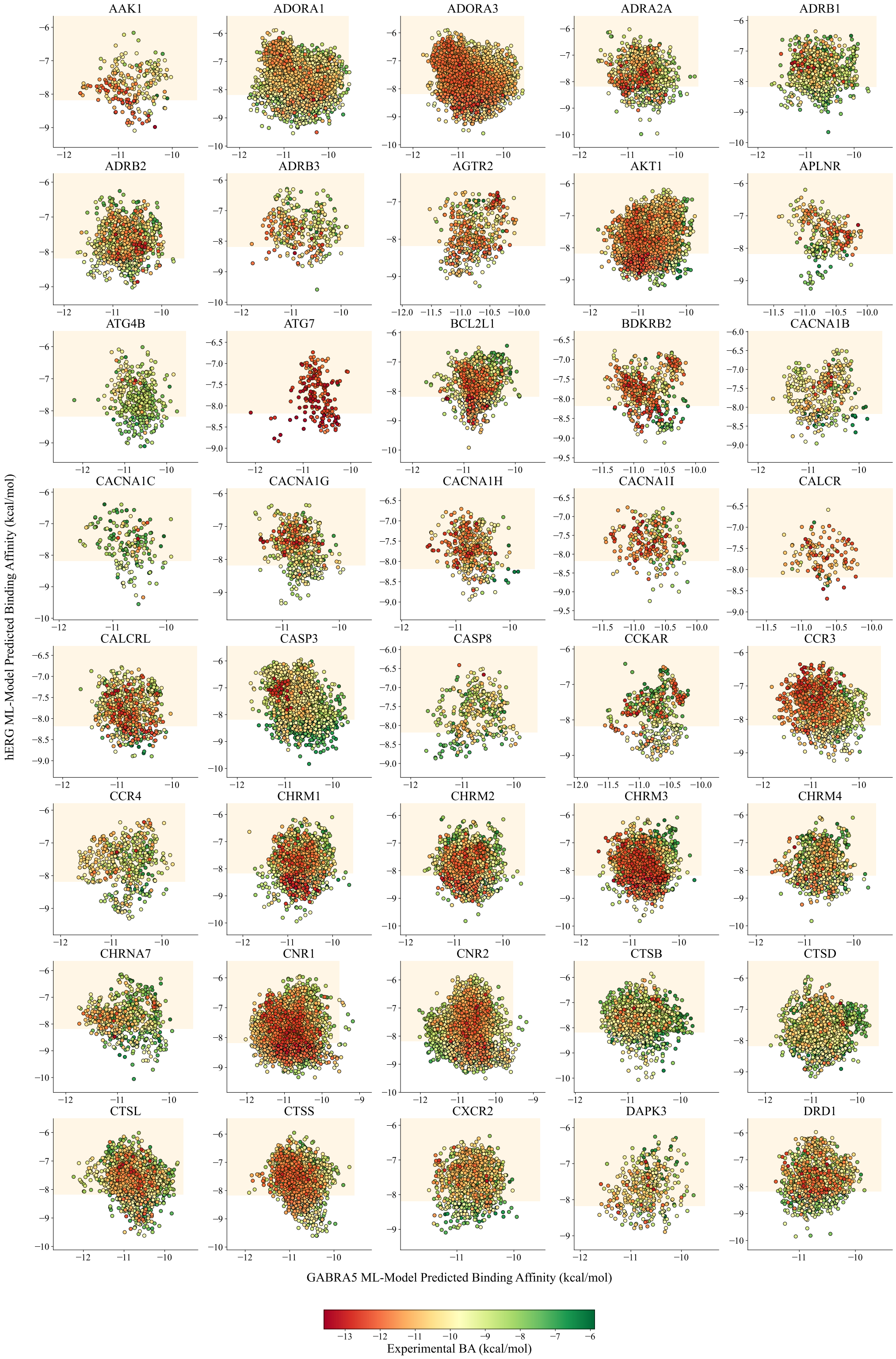

Figure S10: Part A: BA predictions to GABRA5 and hERG from 40 of the 134 inhibitor datasets in our PPI network. The predicted BAs to GABRA5 and hERG are shown on the x- and y-axes, respectively. The orange frames highlight the best regions where the inhibitor has the potential to repurpose GABRA5 and has low side effects on hERG.

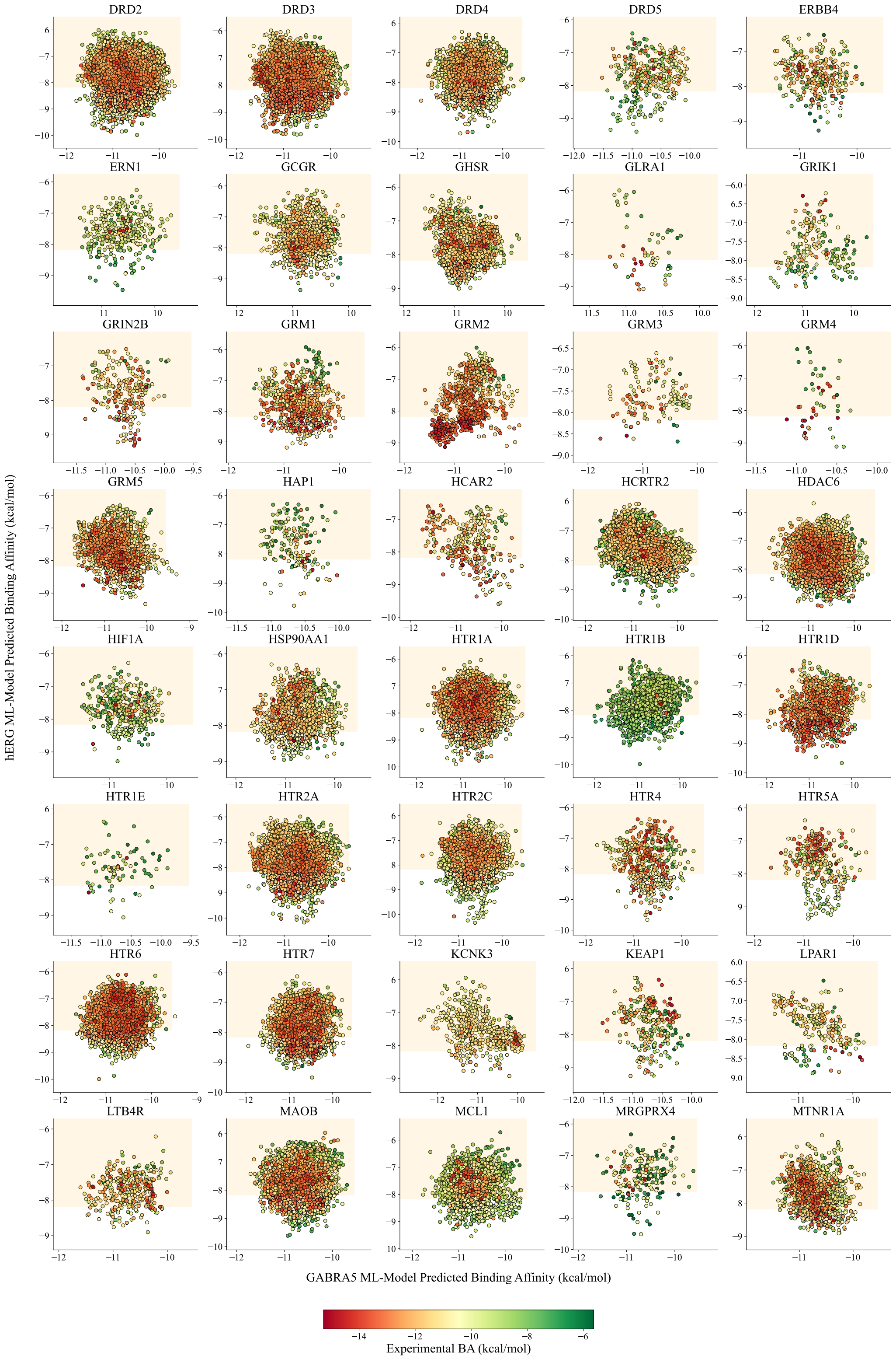

Figure S11: Part B: BA predictions to GABRA5 and hERG from 40 of the 134 inhibitor datasets in our PPI network. The predicted BAs to GABRA5 and hERG are shown on the x- and y-axes, respectively. The orange frames highlight the best regions where the inhibitor has the potential to repurpose GABRA5 and has low side effects on hERG.

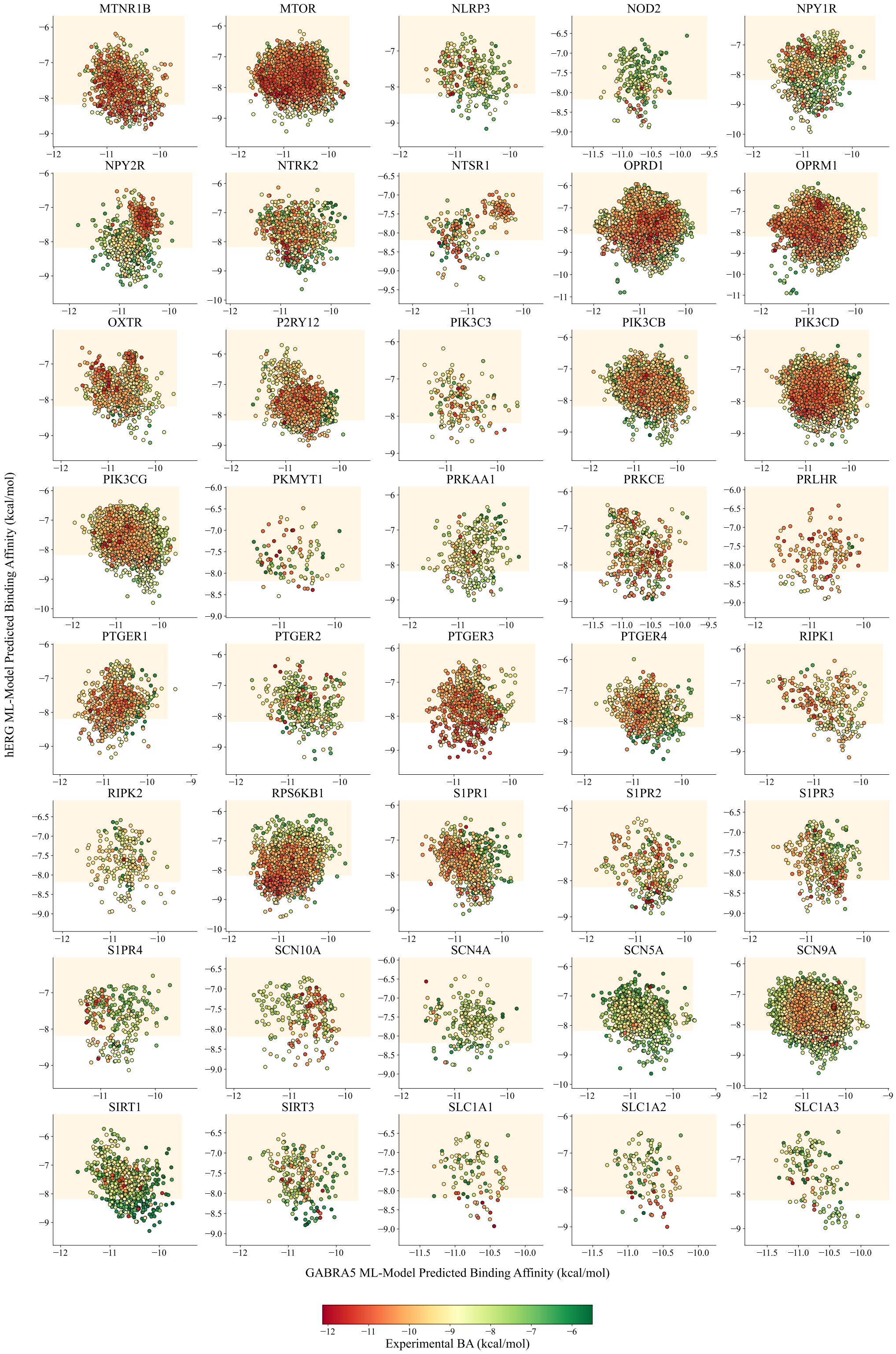

Figure S12: Part C: BA predictions to GABRA5 and hERG from 40 of the 134 inhibitor datasets in our PPI network. The predicted BAs to GABRA5 and hERG are shown on the x- and y-axes, respectively. The orange frames highlight the best regions where the inhibitor has the potential to repurpose GABRA5 and has low side effects on hERG.

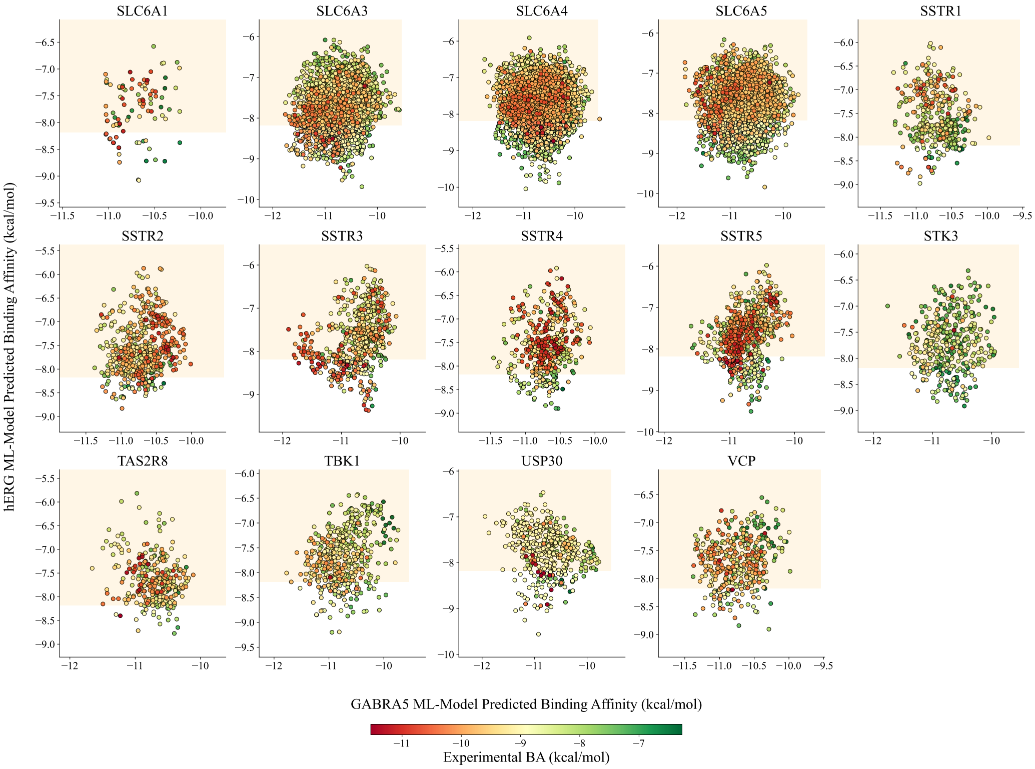

Figure S13: Part D: BA predictions to GABRA5 and hERG from 14 of the 134 inhibitor datasets in our PPI network. The predicted BAs to GABRA5 and hERG are shown on the x- and y-axes, respectively. The orange frames highlight the best regions where the inhibitor has the potential to repurpose GABRA5 and has low side effects on hERG.

mode	affinity (kcal/mol)	dist from rmsd l.b.	best mode rmsd u.b.
1	-8.0	0.000	0.000
2	-7.6	2.111	4.069
3	-7.3	1.231	1.555
4	-7.2	2.963	5.992
5	-7.1	13.748	16.738
6	-7.0	2.973	6.121
7	-6.8	2.501	3.556
8	-6.8	3.697	5.096
9	-6.7	3.269	6.277

Table S10: Details of the docking between ChEMBL200482 and GABRA5.

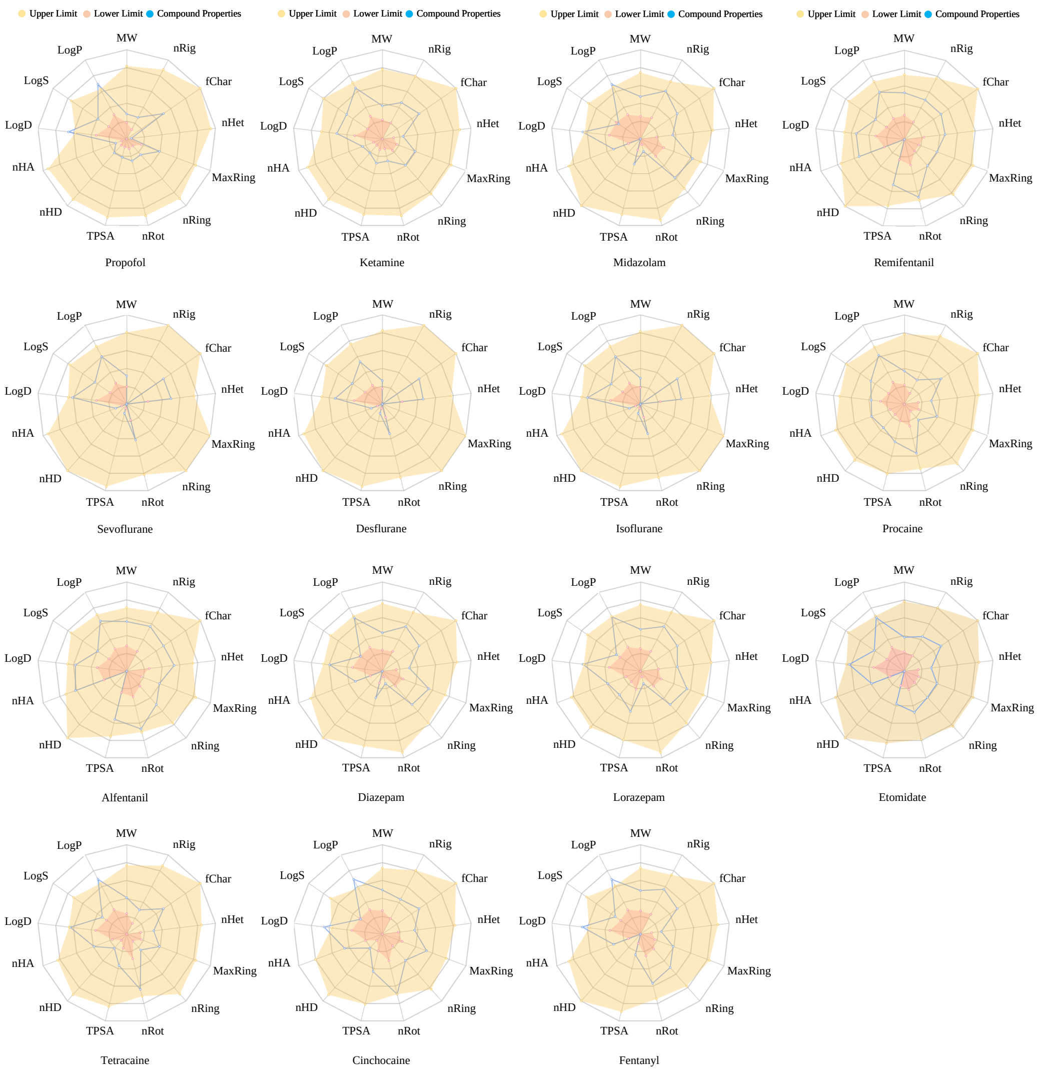

Figure S14: ADMET properties of 15 extant drugs.

	DrugBank ID	Generic name	BA-GABRA5	BA-hERG
1	DB00446	Chloramphenicol	-11.94	-7.37
2	DB12941	Darolutamide	-11.92	-7.69
3	DB06234	Maribavir	-11.68	-7.96
4	DB15091	Upadacitinib	-11.66	-7.95
5	DB17472	Pirtobrutinib	-11.64	-7.26
6	DB06292	Dapagliflozin	-11.63	-8.06
7	DB11179	Exametazime	-11.63	-7.53
8	DB00198	Oseltamivir	-11.61	-7.87
9	DB01190	Clindamycin	-11.55	-7.49
10	DB01066	Cefditoren	-11.55	-6.85
11	DB06654	Safinamide	-11.54	-8.02
12	DB14656	Chlorphenesin carbamate	-11.53	-8.11
13	DB01140	Cefadroxil	-11.53	-6.61
14	DB07565	Chloramphenicol succinate	-11.50	-6.93
15	DB09038	Empagliflozin	-11.47	-7.03

Table S11: A summary of FDA-approved drugs with potential for repurposing as anesthetics, including the binding affinities to GABRA5 and hERG predicted by our machine learning model (measured in kcal/mol). All 15 drugs have binding affinity values for GABRA5 lower than -9.54 kcal/mol, and for hERG, the BA values are all higher than -8.18 kcal/mol.

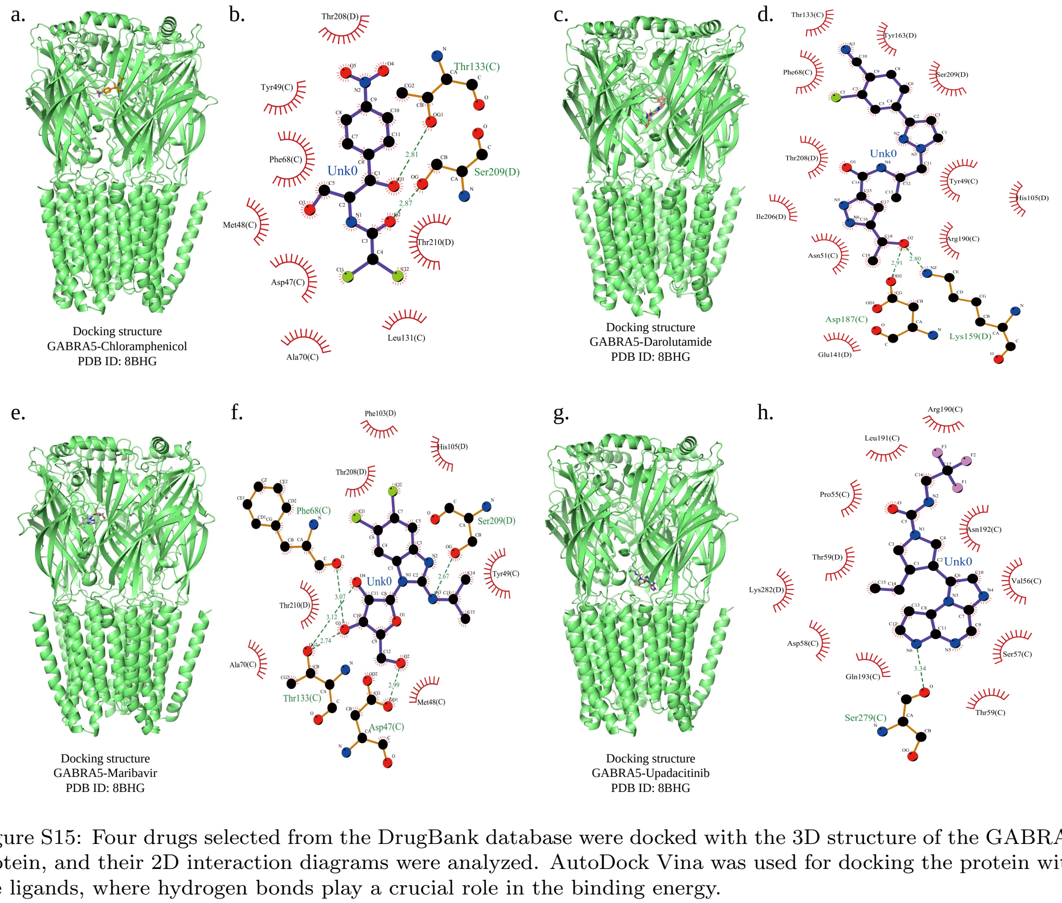

Figure S15: Four drugs selected from the DrugBank database were docked with the 3D structure of the GABRA5 protein, and their 2D interaction diagrams were analyzed. AutoDock Vina was used for docking the protein with the ligands, where hydrogen bonds play a crucial role in the binding energy.

mode	affinity (kcal/mol)	dist from rmsd 1.b.	best mode rmsd u.b.
1	-7.951	0.000	0.000
2	-7.073	1.763	2.106
3	-6.851	19.44	20.49
4	-6.839	25.36	26.73
5	-6.707	27.07	28.53
6	-6.589	19.4	20.47
7	-6.54	5.693	8.704
8	-6.492	26.39	27.88
9	-6.474	27.86	29.34

Table S12: Details of the docking between Chloramphenicol and GABRA5.

mode	affinity (kcal/mol)	dist from rmsd 1.b.	best mode rmsd u.b.
1	-10.45	0.000	0.000
2	-10.1	2.751	4.455
3	-9.85	27.42	30.5
4	-9.72	28.42	31.51
5	-9.412	28.49	31.22
6	-9.164	21.47	26
7	-9.102	21.83	26.33
8	-8.957	19.67	23.31
9	-8.9	23.38	26.69

Table S13: Details of the docking between Darolutamide and GABRA5.

mode	affinity (kcal/mol)	dist from rmsd 1.b.	best mode rmsd u.b.
1	-8.437	0.000	0.000
2	-8.417	2.86	5.181
3	-7.295	32.98	34.6
4	-6.957	19.64	20.75
5	-6.727	4.863	7.616
6	-6.726	26.91	28.47
7	-6.625	21.17	22.06
8	-6.614	19.36	20.21
9	-3.895	27.29	28.34

Table S14: Details of the docking between Maribavir and GABRA5.

mode	affinity (kcal/mol)	dist from rmsd 1.b.	best mode rmsd u.b.
1	-8.97	0.000	0.000
2	-7.769	32.5	34.1
3	-7.363	4.434	6.1
4	-7.247	5.149	8.179
5	-7.17	3.09	3.995
6	-7.037	3.686	5.029
7	-6.997	2.031	2.466
8	-6.858	29.82	32.05
9	-6.835	8.894	10.57

Table S15: Details of the docking between Upadacitinib and GABRA5.

References

[1] L.-N. Chen, W.-W. Wang, Y.-J. Dong, D.-D. Shen, J. Guo, X. Yu, J. Qin, S.-Y. Ji, H. Zhang, Q. Shen, et al. Structures of the endogenous peptide-and selective non-peptide agonist-bound sstr2 signaling

complexes. *Cell Research*, 32(8):785–788, 2022.

[2] E. Chorin, L. Wadhwani, S. Magnani, M. Dai, E. Shulman, C. Nadeau-Routhier, R. Knotts, R. Bar-Cohen, E. Kogan, C. Barbhaiya, et al. Qt interval prolongation and torsade de pointes in patients with covid-19 treated with hydroxychloroquine/azithromycin. *Heart rhythm*, 17(9):1425–1433, 2020.

[3] P. Cook, M. Davies, K. Cronin, and P. Moran. A prospective randomised trial comparing spinal anaesthesia using hyperbaric cinchocaine with general anaesthesia for lower limb vascular surgery. *Anaesthesia and intensive care*, 14(4):373–380, 1986.

[4] A. El Sherbini, K. Liblik, J. Lee, A. Baranchuk, S. Zhang, and M. El-Diasty. Opioids-induced inhibition of herg ion channels and sudden cardiac death, a systematic review of current literature. *Trends in Cardiovascular Medicine*, 2023.

[5] H. Feng and G.-W. Wei. Virtual screening of drugbank database for herg blockers using topological laplacian-assisted ai models. *Computers in biology and medicine*, 153:106491, 2023.

[6] K. Furutani. Facilitation of herg activation by its blocker: A mechanism to reduce drug-induced proarrhythmic risk. *International Journal of Molecular Sciences*, 24(22):16261, 2023.

[7] Y.-g. Li, X.-y. Meng, X. Yang, S.-l. Ling, P. Shi, C.-l. Tian, and F. Yang. Structural insights into somatostatin receptor 5 bound with cyclic peptides. *Acta Pharmacologica Sinica*, pages 1–9, 2024.

[8] N. W. Lonardo, M. C. Mone, R. Nirula, E. J. Kimball, K. Ludwig, X. Zhou, B. C. Sauer, K. Nechodom, C. Teng, and R. G. Barton. Propofol is associated with favorable outcomes compared with benzodiazepines in ventilated intensive care unit patients. *American journal of respiratory and critical care medicine*, 189(11):1383–1394, 2014.

[9] M. L. Ponte, G. A. Keller, and G. D. Girolamo. Mechanisms of drug induced qt interval prolongation. *Current drug safety*, 5(1):44–53, 2010.

[10] K. Sridharan and G. Sivaramakrishnan. Comparison of fentanyl, remifentanil, sufentanil and alfentanil in combination with propofol for general anesthesia: a systematic review and meta-analysis of randomized controlled trials. *Current clinical pharmacology*, 14(2):116–124, 2019.

[11] S. Sugimura, R. Imai, T. Katoh, H. Makino, K. Hokamura, T. Kurita, Y. Suzuki, Y. Aoki, T. Kimura, K. Umemura, et al. Effects of volatile anesthetics on circadian rhythm in mice: a comparative study of sevoflurane, desflurane, and isoflurane. *Journal of Anesthesia*, 38(1):10–18, 2024.

[12] S. Wacker, S. Y. Noskov, and L. L. Perissinotti. Computational models for understanding of structure, function and pharmacology of the cardiac potassium channel kv11. 1 (herg). *Current Topics in Medicinal Chemistry*, 17(23):2681–2702, 2017.

[13] X. Zhang, J. Mao, M. Wei, Y. Qi, and J. Z. Zhang. Hergspred: accurate classification of herg blockers/nonblockers with machine-learning models. *Journal of chemical information and modeling*, 62(8):1830–1839, 2022.

[14] W. Zhao, S. Han, N. Qiu, W. Feng, M. Lu, W. Zhang, M. Wang, Q. Zhou, S. Chen, W. Xu, et al. Structural insights into ligand recognition and selectivity of somatostatin receptors. *Cell Research*, 32(8):761–772, 2022.
